# Ecological Conditions Favoring Budding in Colonial Organisms under Environmental Disturbance

**DOI:** 10.1371/journal.pone.0091210

**Published:** 2014-03-12

**Authors:** Mayuko Nakamaru, Takenori Takada, Akiko Ohtsuki, Sayaki U. Suzuki, Kanan Miura, Kazuki Tsuji

**Affiliations:** 1 Department of Value and Decision Science, Tokyo Institute of Technology, Tokyo, Japan; 2 Graduate School of Environmental Earth Science, Hokkaido University, Sapporo, Japan; 3 The Graduate University for Advanced Studies, Kanagawa, Japan; 4 Faculty of Agriculture, University of the Ryukyus, Okinawa, Japan; American University in Cairo, Egypt

## Abstract

Dispersal is a topic of great interest in ecology. Many organisms adopt one of two distinct dispersal tactics at reproduction: the production of small offspring that can disperse over long distances (such as seeds and spawned eggs), or budding. The latter is observed in some colonial organisms, such as clonal plants, corals and ants, in which (super)organisms split their body into components of relatively large size that disperse to a short distance. Contrary to the common dispersal viewpoint, short-dispersal colonial organisms often flourish even in environments with frequent disturbances. In this paper, we investigate the conditions that favor budding over long-distance dispersal of small offspring, focusing on the life history of the colony growth and the colony division ratio. These conditions are the relatively high mortality of very small colonies, logistic growth, the ability of dispersers to peacefully seek and settle unoccupied spaces, and small spatial scale of environmental disturbance. If these conditions hold, budding is advantageous even when environmental disturbance is frequent. These results suggest that the demography or life history of the colony underlies the behaviors of the colonial organisms.

## Introduction

Dispersal distance varies widely among organisms. Dispersal determines the genetic structure of the meta-population and influences the evolution of individual characteristics such as mortality, reproductive efforts, sex allocation and altruism [1]–[3]. Since dispersal is a costly activity, why organism disperse has become an important issue in evolutionary ecology [4], [5]. In the pioneering meta-population model of Hamilton and May (1977) it was shown that dispersal can evolve even when the dispersal cost is so high that nearly all dispersers are destined to die. Subsequent studies identified specific factors that favor dispersal over non-dispersal, such as the avoidance of competition among relatives and inbreeding [1], [2]. Disturbances such as predation, drought and flooding are regarded as important general triggers of dispersal, encouraging organisms to hedge the risks [6].

However, many organisms do not disperse long distances. For example, some organisms often adopt an “budding” strategy, where a colony splits into two or more parts which subsequently move away from each other. The budding by clones, buds, or propagules has been studied theoretically and experimentally from the viewpoint of the evolution of altruism and sex ratio [7]–[9]. However, those studies did not focus on the difference in dispersal distance, while in many real organisms the budding strategy is typically characterized by shorter-range dispersal in comparison to non-budding dispersal. Contrary to the conventional view, organisms reproducing by budding often survive in environments undergoing frequent disturbance. For example, many invasive clonal plants that are specialists of disturbed habitats predominantly use vegetative reproduction, despite retaining the seed-producing capability that would enhance their spread [10], [11]. New colonies of branching gorgonian coral (*Plexaurella* sp.) exposed to waves are predominantly founded by fragments of broken branches, rather than by inseminated gametes that can migrate long distances [12]. In some invasive ants, such as *Linepithema humile* (the Argentine ant) and *Wasmannia auropunctata* (the tiny fire ant), winged queens do not engage in nuptial (dispersal) flights. Instead they mate within or near natal nests, and new colonies are founded by colony-splitting (fission or budding), in which queens search for a new nest site on foot, accompanied by some workers [13], [14]. These invaders are recognized as specialists of disturbed habitats [13], [15], [16]. However, the ecological conditions favoring budding remain obscure.

All of the above examples (clonal plants, corals and ants) are colonial or super organisms that must maintain a spatially fixed life following initial settlement. Assuming such colonial life, Nakamaru et al. (2007) pioneered the investigation of ecological conditions favoring budding under disturbance in a spatially explicit computational model (lattice model) [17]. They identified an important trade-off between dispersal distance and offspring size (table 1); small reproductive units, such as plant seeds, spawned coral eggs and winged ant queens, travel long-distance, whereas large units, such as the propagules of clonal plants, broken branches of corals and dependently-founded ant colonies remain within their local environment. This difference in dispersal affects the survival and growth of offspring; small offspring suffer low survival and reach maturity comparatively slowly, while large offspring (buds) benefit from high survival and rapid growth to maturity. Nakamaru et al. (2007) concluded that the short distance dispersal strategy (budding) is advantageous over long distance dispersal (non-budding) under conditions of (i) very high mortality of a small-sized colony, or (ii) relatively frequent environmental disturbance over a small spatial scale.

**Table 1:** Life history characteristics of long and short dispersal units.

Dispersal units	Seeds, spawned eggs, and independently founding queens	vegetative propagule, broken coral braches, or dependently-founded ant colonies
Dispersal distance	long	short
Size	small	large
Time to reproduction	long	short
Number	many	few
Survival rate	low	high

However, Nakamaru et al. did not clarify the effect of spatial structure and the life history on the above-mentioned advantage. For each colony, they specifically assumed that growth is logistic and that death rate is a negative exponential function of colony size. However, other survivorship patterns and other growth functions are possible in nature. The authors failed in their quest for a mathematical model that could accommodate relatively complicated assumptions and therefore could not obtain analytical results. These drawbacks have precluded a precise understanding of the adaptive significance of budding. To more generally understand the ecological conditions that induce budding dispersal, here we investigate the characteristics of an organism's life history functions, such as survivorship and colony growth, that favor budding dispersal under disturbance.

We adopt the general view that life history parameters (survival, growth and reproduction functions) directly determine the evolved dispersal strategy [18], [19]. Environmental conditions, such as spatial structure and disturbance, influence the above parameters in a complicated manner through environment vs. phenotype interactions. In this paper we make a simple generalized assumption that is applicable to colonial organisms adopting similar resource allocation strategies. We first focus on colonial life history using a model without spatial structure, called the baseline model. This model assumes discrete colony size, assigning different death and growth probabilities to each colony size. Under this assumption, we can construct discrete equations or a matrix model in the baseline model that permit exact solutions. We clarify the effects of life histories or demographics (such as survivorship and growth rate) on the competition between the two strategies, i.e. budding vs. non-budding. We also examine the conditions under which budding is advantageous over non-budding when realistic and possible assumptions are added to the baseline model.

## Methods and Results

### Model 1: The baseline model in the completely mixed population

It is common that an individual is assumed to be a basic unit in the agent-based simulation model. In contrast, here the basic unit in our agent-based simulation model is the clonal (super) organism or the colony of individuals, and the model is called the colony-based model. Gardner and Grafen (2009) proved that there is no mathematical difference between an individual organism and a clonal group if social groups have no intracolonial conflicts [20].

**Figure 1:**
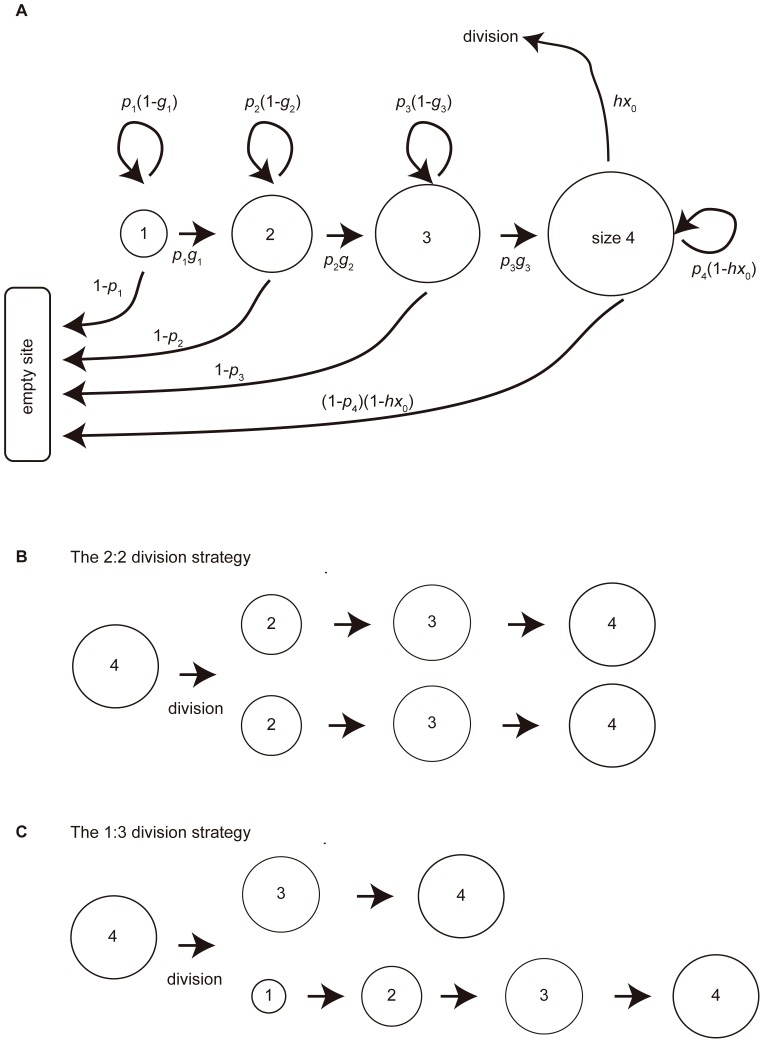
Life history of a colony and its division strategies. (A) Life history of a colony. (B) The 2∶2 division strategy, in which a mature (size 4) always divides into two size 2 daughter colonies. The new colonies grow to maturity. (C) The 1∶3 division strategy, in which a mature colony always divides into a small (size 1) and a large (size 3) daughter colony. The size 1 matures slowly via size 2 and size 3, while the size 3 colony matures rapidly.

We consider only non-spatial structures or completely mixed populations, ignoring the effect of dispersal distance. Instead, we aim to clarify the effect of colonial life history patterns on the population dynamics. The colony size-structured matrix model assumes four colony sizes, designated size 1, size 2, size 3 and size 4. The first three denote immature colonies, while size 4 represents the mature colony. Life history pattern is described by two parameters; the baseline death probability and the growth probability of the colony (Fig. 1 A). The baseline death probability of a colony of size *i* is defined as *d_i_* (0≤*d_i_*≤1, *i* = 1, 2, 3 and 4.). An additional death probability, independent of the baseline death, is imposed by disturbance. The survival probability of a colony of size *i* is 1−*d_i_*, defined as *p_i_* (0≤*p_i_*≤1, *i* = 1, 2, 3 and 4). Let *g_i_* be the probability that a colony of size *i* expands to size *i*+1 (0≤*g_i_*≤1, *i* = 1, 2, 3). When a colony of size *i* survives with probability *p_i_*, it expands to size *i*+1 with probability *g_i_* (*i* = 1, 2, 3); otherwise it remains at its present size with probability 1−*g_i_*. When the colony has reached size 4, it can divide into two colonies, one of which will colonize an empty site. Therefore the chance of division is proportional to the density of empty sites in the population multiplied by the division rate, *h*, where *h* controls the division speed. A size 4 colony deprived of the opportunity to divide will survive with probability *p*_4_.

The colony divides by one of two strategies: designated 2∶2 division and 1∶3 division (Fig.1 B and C). A size 4 colony adopting the 2∶2 division strategy divides into two colonies of size 2, one of which moves to an empty site. Alternatively, a size 4 colony adopting the 1∶3 division strategy divides into two unequally sized colonies (of size 1 and size 3), the smaller of which moves to an empty site. This situation mimics nature; if a biological colony splits into two equal parts, one of the daughter colonies moves to a new site close to the natal colony; if the colony is unequally split, the smaller colony disperses far from the natal colony. The 2∶2 and 1∶3 division strategies correspond to budding and seed-like dispersal, respectively. To purely clarify the effect of division ratio on the population dynamics, we ignore spatial structure and distance.

The size-structured matrix model for colonies adopting the 2∶2 division strategy in the population is constructed as
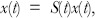
(1)where *x*(*t*)  =  (*x*_0_(*t*), *x*_1_(*t*), *x*_2_(*t*), *x*_3_(*t*), *x*_4_(*t*))*^t^* and
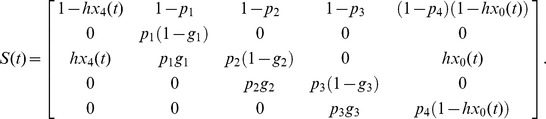



Let *x*_0_(*t*) be the density of empty sites at time *t* and *x_i_*(*t*) (*i* = 1, 2, 3 and 4) be the density of colonies of size *i* adopting the 2∶2 division strategy at time *t*, subject to Σ*_i_ x_i_*(*t*)  = 1. The values of *hx*_4_(*t*) in element (3, 1) and *hx*_0_(*t*) in element (3, 5) show that, when a size 4 colony divides, one of the two resulting size 2 colonies disperses to an empty site while the other remains in its natal habitat.

Similarly, the size-structured matrix model for colonies adopting the 1∶3 division strategy in the population is constructed as
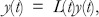
(2)where *y*(*t*)  =  (*y*_0_(*t*), *y*_1_(*t*), *y*_2_(*t*), *y*_3_(*t*), *y*_4_(*t*))*^t^* and
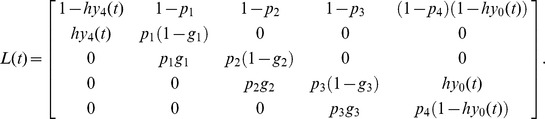



Let *y*_0_(*t*) be the density of empty sites at time *t* and *y_i_*(*t*) (*i* = 1, 2, 3 and 4) be the density of colonies of size *i* adopting the 1∶3 division strategy at time *t*, subject to Σ*_i_ y_i_*(*t*)  = 1. When a size 4 colony divides, the probability of the resulting size 1 colony successfully migrating to an empty site is determined by *hy*_4_(*t*) in element (2, 1), while the probability that the size 3 colony remains in its natal habitat is *hy*_0_(*t*) in element (4, 5).

The dynamics of competition between colonies adopting 1∶3 and 2∶2 division strategies can be described using the competition matrix, *F*(*w*(*t*)):
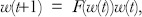
(3)in which the vector *w*(*t*) consists of *x_i_*(*t*) and *y_i_*(*t*) (*i* = 1, 2, 3, 4) and *z*_0_(*t*) which is the density of empty sites, and the matrix *F* represents the colony size transition. Detailed information about Eq. (3) is given in Appendix S4.

### Results when all growth probabilities are one

#### Mathematical results of the colony size-structured matrix model

Here we consider the simplest case in which all growth probabilities are one (*g_i_* = 1). By local stability analysis of Eq. (1), the equilibrium point where colonies adopting the 2∶2 division strategy become extinct (*x*_0_*(*t*)  = 1.0) is locally unstable when
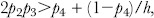
(4)otherwise the equilibrium point *x*_0_*(*t*)  = 1.0 is locally stable (see appendix S1 and S2-(i)). This result implies that colonies adopting the 2∶2 division strategy could stably survive in the local environment under conditions satisfying the inequality (4). Inequality (4) is called the viability condition of the 2∶2 division strategy. When *p*_1≤_ *p*_2≤_ *p*_3≤_ *p*_4_, inequality (4) is locally stable at the equilibrium *x*_1_*(*t*)+*x*_2_*(*t*)+*x*_3_*(*t*)+*x*_4_*(*t*)>0 and *x*_0_*(*t*)<1.0 (see appendix S1 and S2-(ii)).

Similarly, by local stability analysis of Eq. (2), the equilibrium point where colonies adopting the 1∶3 division strategy become extinct (*y*_0_*(*t*) = 1.0) is locally unstable when
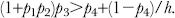
(5)


Under this condition, colonies adopting the 1∶3 division strategy can stably survive; hence, inequality (5) is called the viability condition of the 1∶3 division strategy (appendix S1 and S3-(i)). When *p*_1≤_ *p*_2≤_ *p*_3≤_ *p*_4_, inequality (5) defines the locally stable condition at equilibrium (*y*_1_*(*t*) + *y*_2_*(*t*) + *y*_3_*(*t*) + *y*_4_*(*t*) > 0 and *y*_0_*(*t*) < 1.0) (see appendix S1 and S3-(ii)).

We also analyze the matrix model in which two strategies compete for empty sites (see appendix S4). The notation of parameters and variables is unaltered, apart from the density of empty sites at time *t*, denoted *z*_0_(*t*). As shown in appendix S4-(ii), the equilibrium point of the pure 2∶2 division strategy, (*x*_1_*(*t*) + *x*_2_*(*t*) + *x*_3_*(*t*) + *x*_4_*(*t*) > 0 and y_i_*(*t*)  =  0 (*i* = 1–4)), is locally stable when inequalities (4) and (6) are satisfied, where (6) is given by
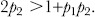
(6)


Appendix S4-(iii) also shows that the equilibrium point of the pure 1∶3 division strategy, (*y*_1_*(*t*) + *y*_2_*(*t*) + *y*_3_*(*t*) + *y*_4_*(*t*) > 0 and x_i_*(*t*)  = 0 (*i* = 1–4)), is locally stable when inequalities (5) and (7) are satisfied, where (7) is given by
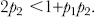
(7)


Inequalities (6) and (7) are called the competition conditions.

These analyses indicates that the 2∶2 division strategy wins against the 1∶3 division strategy when inequalities (4) and (6) are satisfied, while upholding inequalities (5) and (7) favors the 1∶3 division strategy. Inequalities (4)–(7) indicate that the division rate *h* does not affect the competition condition, but does affect the viability conditions. Under the viability conditions, small *h* restrains colony survivorship. The competition conditions are influenced by the survival probabilities of colonies of size 1 and 2 (see inequalities (6) and (7)), but not by those of older colonies.

Equation (6) can be rewritten as *p*_2_>1/(2−*p*_1_)≥1/2, indicating that colonies adopting the 2∶2 division strategy can overcome their 1∶3 counterparts when *p*_2_ exceeds 0.5. Inequality (7) can be rewritten as *p*_2_<1/(2−*p*_1_)≤1. Equations (6) and (7) also indicate that relatively high *p*_2_ can favor colonies adopting the 2∶2 division strategy.

When *d*_1_ = *d*_2_ = *d*_3_ = *d*_4_, i.e. the dynamics are size-independent, inequality (7) always holds. This result implies that, in size-independent dynamics, the 1∶3 division strategy is always advantageous over the 2∶2 division strategy.

To investigate whether inequalities (4) – (7) hold for any values of *p*_1_, *p*_2_, *p*_3_ and *p*_4_, we conduct the numerical simulations of Eq. (D1) presented in appendix S4, and also run the colony-based simulations.

#### Simulation results of competition for empty sites between two reproductive strategies (2∶2 division and the 1∶3 division)

We conducted colony-based simulations based on the assumptions in the previous section. We assume 2,500 sites, either unoccupied or occupied by colonies adopting one of two reproductive strategies. Each parameter set was run 100 times.

Three forms of the baseline death probability function are considered in the simulations. Death type (i) assumes that *d*_2_ = *d*_3_ = *d*_4_. If *d*_1_ is higher than the other rates, this function is similar to the exponential decreasing function adopted in Nakamaru et al. [17]. In death type (ii), the death probability function is a linear function of size, and defining *d*_1_ and *d*_4_ automatically determines *d*_2_ and *d*_3_. In death type (iii), we assume that *d*_1_ = *d*_2_ = *d*_3_. If *d*_1_ = *d*_4_, the four death probabilities are identical. In this scenario, we can investigate the size-independent ecological dynamics and compare them with the size-dependent dynamics.

**Figure 2:**
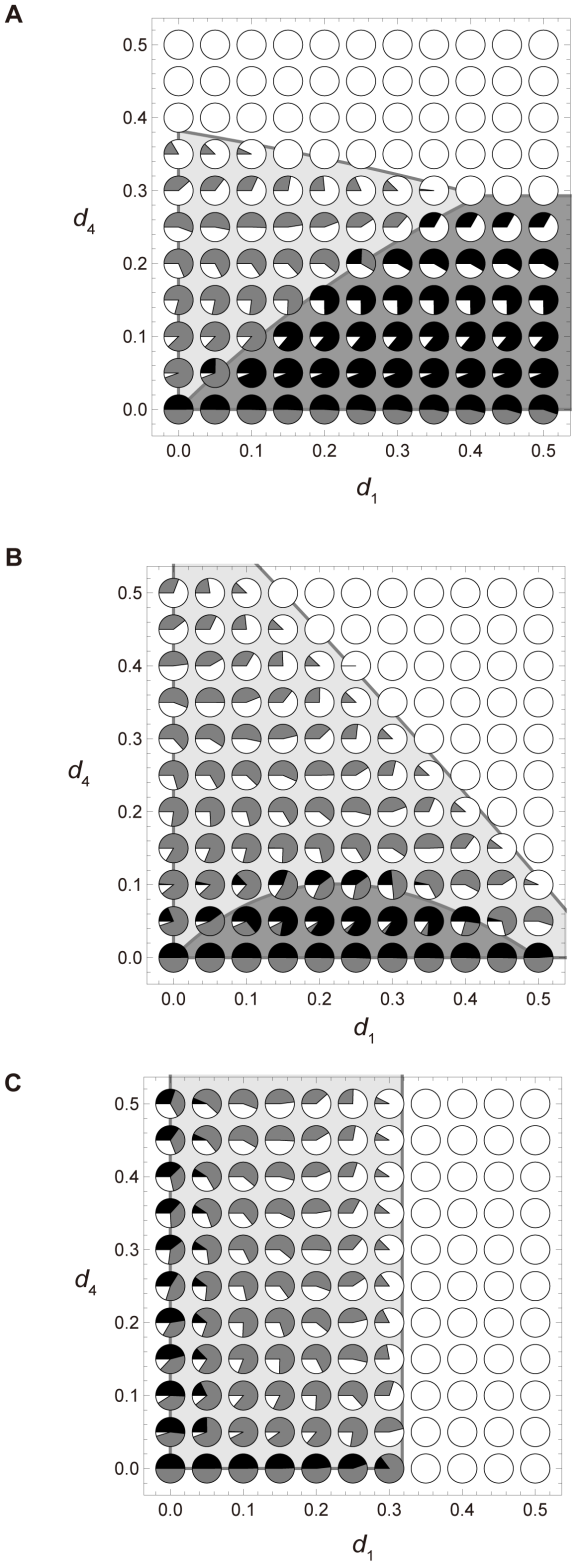
The *d*_1_-*d*_4_ graphs of the theoretical and simulated solutions in a completely mixed population. The horizontal and vertical axes represent *d*_1_ and *d*_4_, respectively The lines in the graphs delineate the viability and competition conditions in the completely mixed population. Theoretical analysis predicts that the 2∶2 division strategy wins in the dark gray area, while the 1∶3 division strategy wins in the light gray area. Each pie chart shows the average densities of sites occupied by either of the two strategies, as well as the empty sites (open circles) after 10,000 iterations in 100 simulation runs. The black and gray portions of the filled circles indicate the average density of sites occupied by the 2∶2 and 1∶3 division strategies, respectively. In *d*_4_ = 0.0, matured colonies adopting both strategies ultimately survive and neutrally coexist. Initial densities are *z*_0_ = 0.2 and *x*_1_ = *x*_2_ = *x*_3_ = *x*_4_ = *y*_1_ = *y*_2_ = *y*_3_ = *y*_4_ = 0.1. Other parameters are: 2,500 sites in the population, *u* = 0 and *h* = 1. (A) Death probability function assumes that *d*_2_ = *d*_3_ = *d*_4_. (B) Death probability function is linear. (C) Death probability function assumes that *d*_1_ = *d*_2_ = *d*_3_.

To understand the effect of colony division and death probability on the ecological dynamics described by Eqs. (1), (2) and (D1), the theoretical results (computed from inequalities (4) – (7)) and the simulation results are plotted in Fig. 2. We now compare the results of inequalities (4) – (7) with those of the colony-based simulations and the numerical calculations of eq. (3), and show that these approaches are consistent. Figure 2 A shows how the outcomes are affected by two death probabilities (*d*_1_ and *d*_4_) in death type (i). Each parameter set was tested in 100 simulation runs. Since the simulated and theoretical outcomes are consistent, we conclude that inequalities (4) – (7) can predict the simulated ecological dynamics even though *p*_1≤_ *p*_2≤_ *p*_3≤_ *p*_4_ is violated (see appendix S2). When inequality (7) is satisfied (roughly, *d*_1≤_ *d*_4_), colonies adopting the 1∶3 division strategy dominate the population. Conversely, if inequality (6) is upheld (roughly, *d*_1_>*d*_4_), the population is dominated by colonies undergoing 2∶2 division. When *d*_4_ is high or neither viability condition is satisfied, size 4 colonies die out before dispersing their daughter colonies and all colonies become extinct.

Figure 2 B shows the simulation outcomes in the *d*_1_- *d*_4_ graph in death type (ii). In parameter sets satisfying *d*_1_>*d*_4_ and small *d*_4_, colonies adopting the 2∶2 division strategy dominate the population in some cases. When *d*_1_ or *d*_4_ is high, all colonies become extinct. Otherwise, the population is dominated by colonies adopting the 1∶3 division strategy. As also shown in Fig. 2 B, inequalities (4) – (7) can predict the simulated ecological dynamics even when *p*_1≤_ *p*_2≤_ *p*_3≤_ *p*_4_ is violated (See appendix S2). In death type (iii), colonies adopting the 2∶2 division strategy are always overwhelmed by those undertaking the 1∶3 division strategy (figure 2 C).

Figure 2 displays the outcomes of special cases of the death functions. One fixed (*d*_4_ = 0.2) and three variable (*d*_1_, *d*_2_ and *d*_3_) death probabilities were assumed in the additional simulations (figures are not shown). The outcome of this scenario also support inequalities (4) – (7), in which the competition between the two strategies depends on both *d*_1_ and *d*_2_, and colony survivorship is affected by all death probabilities. The high *d*_1_ ensures that colonies adopting the 2∶2 division strategy ultimately win, while low *d*_2_ favors the 2∶2 division strategy.

We conclude that the 2∶2 division strategy is advantageous over the 1∶3 strategy when the death probability of size 1 colonies is relatively higher than that of size 2 colonies (see Eq. (6) and Fig. 2). Otherwise, the 1∶3 strategy is favorable. The reasons for these findings are now discussed: Fig. 1 C shows that the 1∶3 strategy divides a mature colony into two daughter colonies of unequal size (1 and 3). The size 1 colony matures much more slowly than the size 3 colony, which rapidly (by comparison) reaches size 4 and divides. By contrast, the 2∶2 strategy divides a mature colony into two half-sized colonies (figure 1 B). A size 2 colony matures more rapidly than a size 1 colony but less rapidly than one of size 3. Therefore, if all colonies share the same death probability, the 1∶3 division strategy is advantageous over the 2∶2 strategy because the probability of reproduction is higher for size 3 colonies than for size 2 colonies (see Fig. 1 B and C).

Now consider the scenario in which the death probability of a size 1 colony is prohibitively high. Once colonies adopting the 1∶3 division strategy have divided, the size 1 daughter colonies die out, while the size 3 colonies survive and mature. Colonies adopting the 2∶2 division strategy are not affected by the small survival chance of size 1 colonies, and both daughter colonies will likely survive.

Next, we assign a high death probability to a size 2 colony, and observe the effects on the ecological dynamics. Figure 1 B indicates that colonies adopting the 2∶2 division strategy can scarcely increase their numbers because most of the daughter colonies dies out before reaching maturity. Meanwhile, although new size 1 colonies also die out, the size 3 colonies can survive to maturity. Therefore, imposing a high death probability on size 2 colonies favors colonies adopting the 1∶3 division strategy. This discussion is supported by the competition conditions (Eqs. (6) and (7)).

### The results when all growth probabilities are less than one

The following analysis concerns the effect of growth probability on the ecological dynamics. Applying the methods of appendices S1–S3 to Eqs. (1) and (2), the viability condition of the 2∶2 and 1∶3 division strategies are respectively obtained as
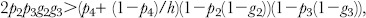
(8)

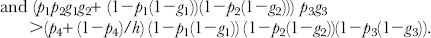
(9)


Applying the methods of appendix S4 to Eq. (3) and to the general equation (D1), we can calculate the conditions under which the 2∶2 division strategy wins against the 1∶3 strategy when inequalities (8) and (10) are satisfied, where (10) is given by
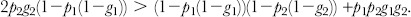
(10)


On the other hand, if inequalities (9) and (11) hold, the 1∶3 division strategy can defeat the 2∶2 strategy, where (11) is given by
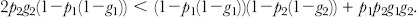
(11)


Inequalities (10) and (11) are called the competition conditions.

The competition conditions are independent of the division rate *h*, but *h* does affect the viability conditions. In essence, small *h* restrains colony survival. The competition conditions are influenced by the survival and growth probabilities of size 1 and 2 colonies (see also inequalities (6) and (7)), but not by those of older colonies.

When *d*_1_ = *d*_2_ = *d*_3_ = *d*_4_ and *g*_1_ = *g*_2_ = *g*_3_, interpreted as no colony dynamics, inequality (11) is always upheld. This result implies that, in static colonies, the 1∶3 division strategy always confers survival at the expense of the 2∶2 division strategy.

**Figure 3:**
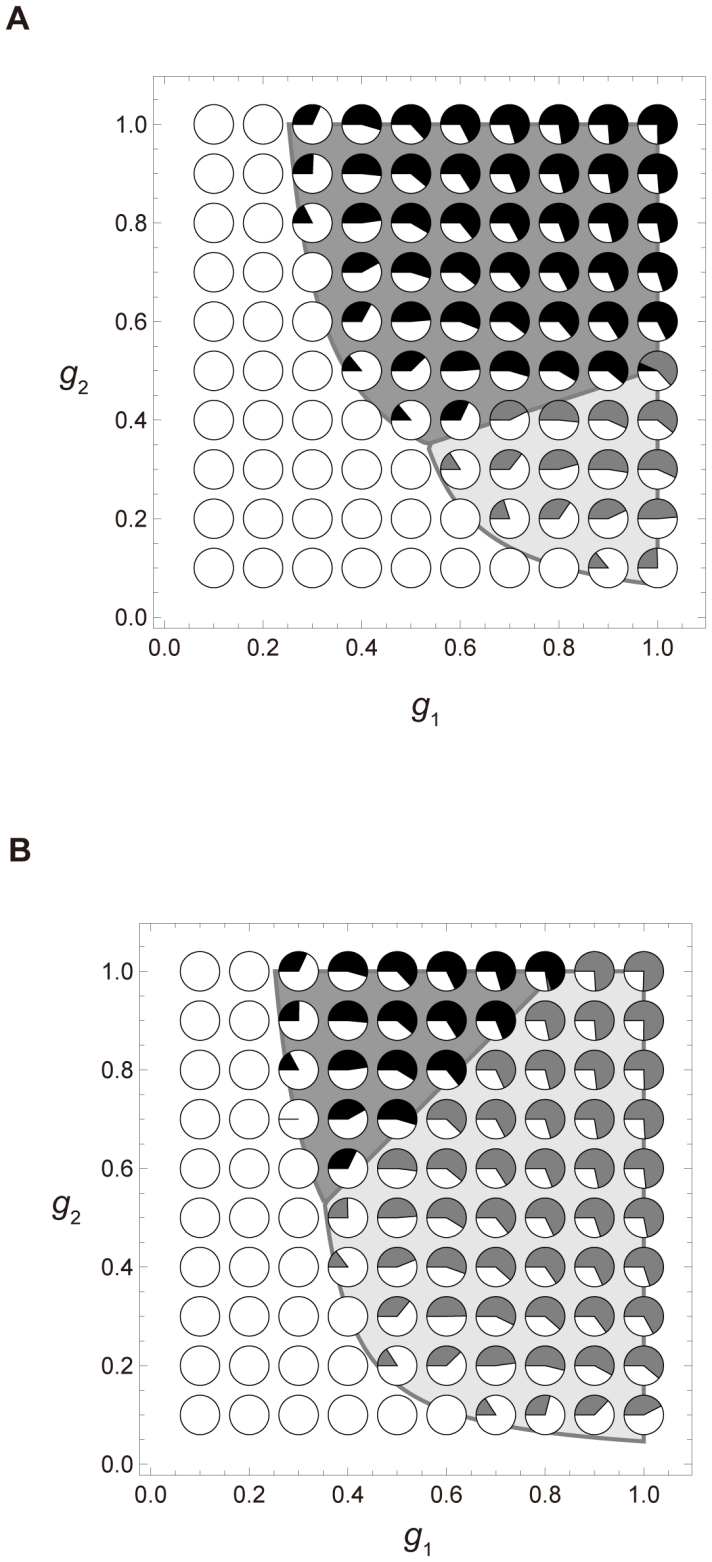
The *g*_1_-*g*_2_ plot of simulated and theoretical results in a completely mixed population. Simulations are iterated 6,250 times. Parameters are *d*_2_ = *d*_3_ = *d*_4_ = 0.15, *h* = 1, *u* = 0. The horizontal and vertical axes represent *g*_1_ ( = *g*_3_) and *g*_2_, respectively. Initial densities are *z*_0_ = 0.6 and *x*_1_ = *x*_2_ = *x*_3_ = *x*_4_ = *y*_1_ = *y*_2_ = *y*_3_ = *y*_4_ = 0.05. More information is provided in the caption to Fig. 2. (A) *d*_1_ = 0.35. (B) *d*_1_ = 0.15.

We now compare the results of inequalities (8)-(11) with those of the colony-based simulations and the numerical calculations of eq. (3), and show that these approaches are consistent. First, we examine the effect of growth probabilities on the system using the outcomes derived from inequalities (8) – (11). Figure 3 shows the effect of *g*_2_ and *g*_1_ ( = *g*_3_) on the competition and viability conditions defined in inequalities (8) – (11). If *g*_2_>*g*_1_ = *g*_3_, the fastest growing colonies are those increasing from size 2 to 3. This scenario mimics logistic growth. When *d*_1_ = 0.35>*d*_2_ = *d*_3_ = *d*_4_ = 0.15, and also *g*_1_ = *g*_2_ = *g*_3_ = 1 (see Figures 2 A and 3 A), the population is dominated by colonies adopting the 2∶2 division strategy. When *g*_2_>*g*_1_ = *g*_3_, colonies adopting the 2∶2 division strategy can always defeat their unequally dividing counterparts. Conversely, lower *g*_2_ favors colonies adopting the 1∶3 division strategy. When *d*_1_ = *d*_2_ = *d*_3_ = *d*_4_ = 0.15, and also *g*_1_ = *g*_2_ = *g*_3_ = 1, the 1∶3 division strategy becomes favorable (see figure 3 B). When *g*_2_<*g*_1_ = *g*_3_, colonies adopting the 1∶3 division strategy outcompete those adopting the 2∶2 division strategy. The reverse occurs when *g*_2_>*g*_1_ = *g*_3_, especially when *g*_2_ is high. According to Figure 3, low and high *g*_2_ promote the 1∶3 division strategy and the 2∶2 division strategy, respectively. These results are supported by the simulation outcomes.

### Model 2 and results: Direct competition between a dispersing colony and settled colonies

Thus far, the model has assumed no direct competition between colonies; dispersing colonies avoid sites occupied by existing colonies, and seek unoccupied sites in which to settle. However, direct competition is the norm in nature. In this section, we examine the effect of direct competition imposed by dispersing colonies settling at new sites.

Competition is introduced to the baseline model with *g_i_* = 1. Once a colony has divided into two, one of the daughter colonies migrates from the natal colony site to a new site chosen randomly from the site population. If the site is empty, the dispersing colony settles there. If the site is occupied by another colony, the dispersing colony competes with the settled colony for the space. Let *w_ij_* be the probability that a dispersing colony of size *i* invades and annihilates a settled colony of size *j*. We investigate three types of direct competition: (i) *w_ij_* = 0.0, (ii) *w_ij_* = 1.0, (iii) *w_ij_* = 1 if *i*>*j*, *w_ij_* = 0.5 if *i* = *j*, and *w_ij_* = 0.0 if *i*<*j*. In situation (i), the settled colony can overcome the dispersing colony regardless of its size, and the dispersing colony dies out. In (ii), the dispersing colony always combats the settled colony, the settled colony dies out, and the dispersing colony occupies the site. Situation (iii) implies that a larger colony always overtakes a smaller one.

If all colonies in the population adopt the 2∶2 division strategy, the matrix construct is
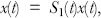
(12)where *x*(*t*)  =  (*x*_0_(*t*), *x*_1_(*t*), *x*_2_(*t*), *x*_3_(*t*), *x*_4_(*t*))*^t^* and
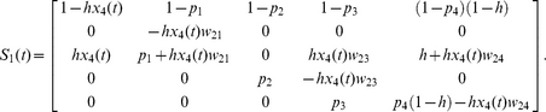



At equilibrium, x*  =  (1, 0, 0, 0, 0)*^t^* is locally unstable when 2*p*_2_ *p*_3_ > *p*_4_ + (1−*p*_4_)/*h* regardless of *w_ij_*, which corresponds to inequality (4).

Similarly, if all colonies in the population adopt the 1∶3 division strategy, the matrix construct is
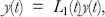
(13)where *y*(*t*)  =  (*y*_0_(*t*), *y*_1_(*t*), *y*_2_(*t*), *y*_3_(*t*), *y*_4_(*t*))*^t^* and
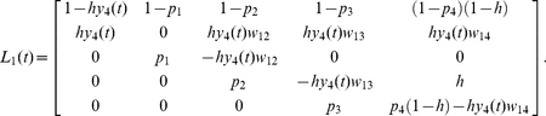



At equilibrium, y*  =  (1, 0, 0, 0, 0)*^t^* is locally unstable when (1+*p*_1_ *p*_2_)*p*_3_ > *p*_4_ + (1−*p*_4_)/*h* regardless of *w_ij_*, which corresponds to inequality (5).

Interestingly, the baseline model with *g_i_* = 1 and the direct competition model share the same local stability condition; an equilibrium in which all colonies become extinct. This arises because a newly-divided dispersing colony encountering many empty sites at equilibrium will more likely settle at a site not already occupied. In this situation, direct competition exerts little influence on the population dynamics.

**Figure 4:**
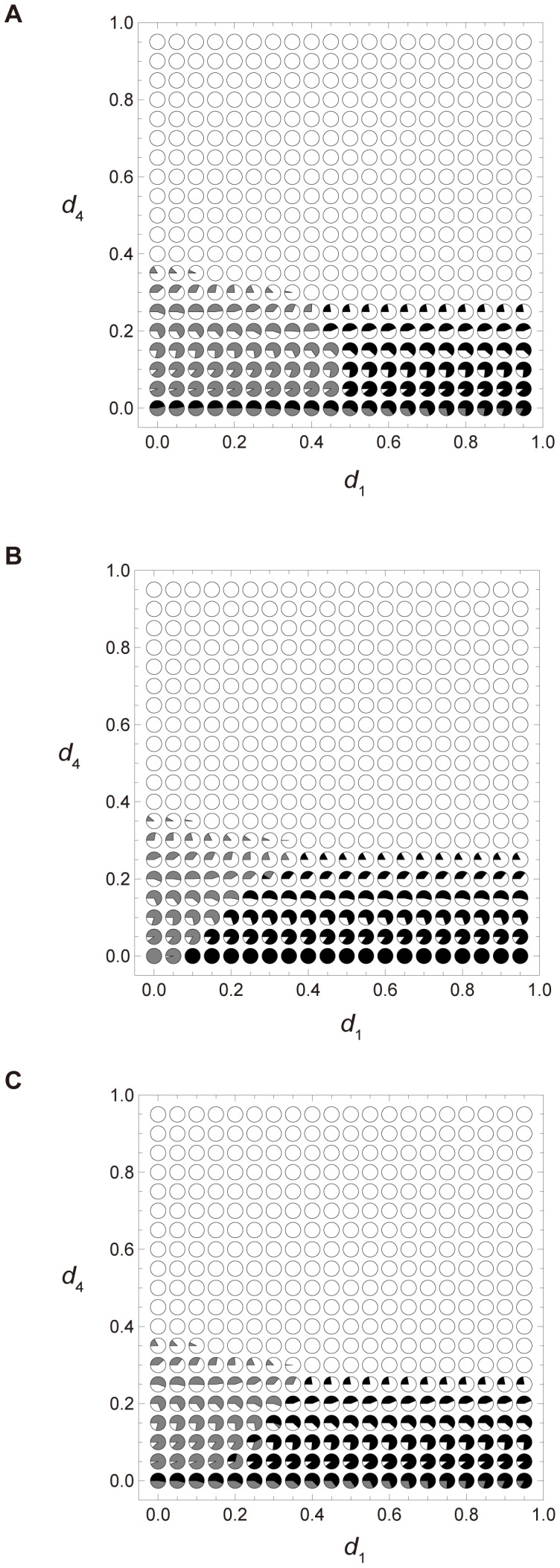
The *d*_1_-*d*_4_ graph of the colony-based simulations in which a dispersing colony competes with settled colonies. The horizontal and vertical axes represent *d*_1_ and *d*_4_, respectively. Death type (i), in which *d*_2_ = *d*_3_ = *d*_4_, is assumed. Each pie chart shows the average densities of sites occupied by either of the two strategies, as well as the empty sites (open circles) after 5,000 iterations of (A) and (B) and after 20,000 iterations of (C) in 100 simulations. The black and gray portions of each circle indicate the average densities of sites occupied by colonies undertaking the 2∶2 division strategy and the 1∶3 division strategy, respectively. The open portion represents the average density of empty sites. Initial densities are *z*_0_ = 0.6 and *x*_1_ = *x*_2_ = *x*_3_ = *x*_4_ = *y*_1_ = *y*_2_ = *y*_3_ = *y*_4_ = 0.05. The other parameters are: 2,500 sites, *u* = 0 and *h* = 1. (A) Direct competition (i), in which *w_ij_* = 0.0. (B) Direct competition (ii), in which *w_ij_* = 1.0. (C) Direct competition (iii), in which a bigger colony always combats a smaller one.

To investigate the effect of direct competition among colonies adopting either reproductive strategy, we run the colony-based simulations under death type (i), which most strongly favors the 2∶2 division strategy among the three death categories. As shown in Figure 4, among the three categories of direct competition, the 2∶2 division strategy is most favored in category (ii).

Next, we discuss why direct competition category (ii), rather than category (i), confers advantage to the 2∶2 division strategy, focusing on the disadvantage of the 1∶3 division strategy in this scenario. The 1∶3 division strategy disperses a size 1 colony, while the larger colony remains at the site. Recall that, in this strategy, a size 3 colony matures more rapidly than a colony of size 1. In direct competition category (ii), a size 3 colony dies out, whereas a size 1 colony disperses and settles at a new site. Since a size 1 colony matures more slowly than a size 2 colony, the 2∶2 division strategy is advantageous over the 1∶3 division strategy. Conversely, in direct competition category (i), a size 3 colony can survive but a size 1 colony cannot colonize a new site. In this scenario, the 1∶3 division strategy is preferable.

The assumptions of the baseline model and direct competition category (i) are similar in that dispersing colonies can settle only at empty sites. However the two scenarios generate completely different outcomes (see Figures 2 A and 4 A). In Figure 2 A, the 2∶2 division strategy is more advantageous than in Figure 4 A, because dispersing colonies always die out under direct competition (i), unless they find an unoccupied site to colonize. Conversely, in the baseline model, colonies do not disperse unless empty sites are available. Although the dispersing colonies die out, the 1∶3 division strategy confers less damage than the 2∶2 division strategy because the size 3 colonies remaining at the natal site after division compensate for the loss of the smallest colonies in direct competition category (i). Populations of colonies adopting the 2∶2 division strategy lose their dispersing size 2 members, which is more damaging to the population dynamics than the loss of size 1 colonies. Therefore the 2∶2 division strategy cannot outcompete the 1∶3 division strategy in this scenario.

Although direct competition (ii) most favors the 2∶2 division strategy among the three direct competitions, it disadvantages the 2∶2 division strategy relative to the baseline model with *g_i_* = 1 (See Figures 2 A and 4 B). Therefore we conclude that the 2∶2 division strategy is advantageous if newly-divided colonies disperse solely to empty sites and never directly compete with colonies to claim an occupied site. In the following section, we investigate the effect of spatial structure and environmental disturbance on the population dynamics by imposing spatial structure on the baseline model.

### Model 3 and results: Spatial structure and environmental disturbance

In a previous section, we found that imposing death type (i) and the logistic growth of colonies favor the 2∶2 division strategy over the 1∶3 strategy when dispersing colonies seek and settle at unoccupied spaces in a completely mixed population. In this section, we examine the environmental factors that favor the 2∶2 division strategy over the 1∶3 division strategy. To this end, we use the lattice-structured population as a spatial structure, assuming death type (i) and that a dispersing colony seeks and settles at an unoccupied site. Although the 2∶2 division strategy is advantageous when *g*_2_>*g*_1_ and *g*_3_, here we set *g*_1_ = *g*_2_ = *g*_3_ = 1 for simplicity. We then run simulations to investigate how the population dynamics are affected by spatial structure and environmental disturbance.

We first present the assumptions of the spatial structured model without environmental disturbance. We use a lattice model as a spatial structure model. Each colony distributed in each lattice site, possesses two traits; colony division and dispersal distance. Each colony adopts either the 2∶2 or the 1∶3 division strategy (Figure 1), and disperses either short- or long-distance.

This setup yields four possible division/dispersal strategies: 1∶3 long, 1∶3 short, 2∶2 long and 2∶2 short. The 1∶3 long strategy disperses the size 1 colony to a random site in the lattice-structured population. The 1∶3 short strategy displaces the size 1 colony to an empty site among the nearest neighbors. The 2∶2 long disperses one of the size 2 colonies to a random site in the lattice-structured population. The 2∶2 short strategy and deposits one of the divided size 2 colonies in a nearest-neighbor empty site. Among these strategies, the 1∶3 long and 2∶2 short are most commonly found in nature. Thus, we focus on the competition between the 1∶3 long and 2∶2 short strategies in the lattice-structured population.

#### Simulated results in the absence of environmental disturbance

**Figure 5:**
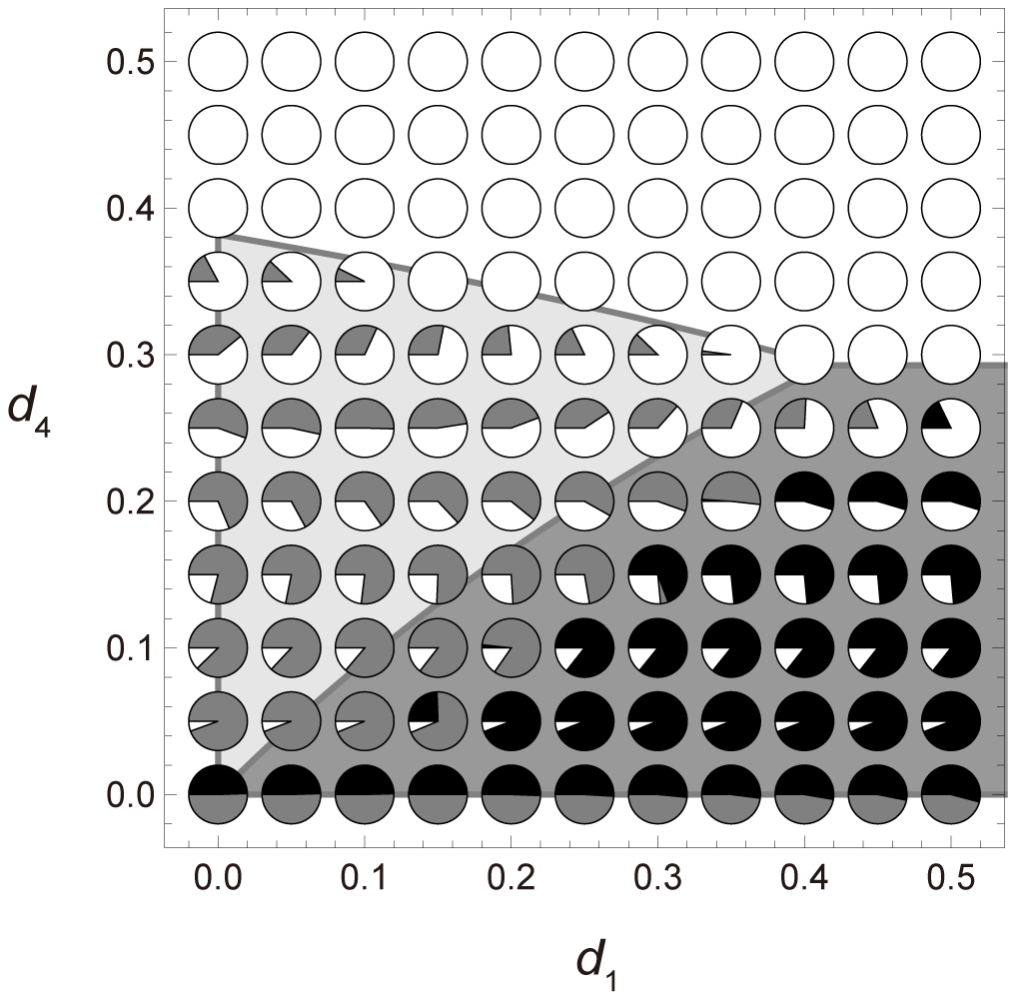
The *d*_1_-*d*_4_ graph of the two-dimensional lattice-structured population. The horizontal and vertical axes represent *d*_1_ and *d*_4_, respectively. Each circle shows the average densities of sites occupied by either of the two strategies, as well as the empty sites (open circles) after 10,000 iterations in 100 simulations on the two-dimensional lattice. The black and gray portions of each circle indicate the average density of the sites occupied by colonies undertaking the 2∶2 division and 1∶3 division strategies, respectively. The open portion represents the average density of the empty sites. The lines in the graphs delineate the theoretical solutions in a completely mixed population. In the dark gray area, the 2∶2 division strategy theoretically wins over the 1∶3 strategy. In the light gray area, the predicted winning strategy is 1∶3. In *d*_4_ = 0.0, mature colonies always survive and a neutral relationship develops between the strategies, which then coexist. Initial densities are *z*_0_ = 0.2 and *x*_1_ = *x*_2_ = *x*_3_ = *x*_4_ = *y*_1_ = *y*_2_ = *y*_3_ = *y*_4_ = 0.1. The other parameters are: 2,500 lattice sites, *u* = 0 and *h* = 1. The death probability assumes death type (i).

First, to clarify the effect of the lattice-structured population on the competition between the two strategies, we assume no environmental disturbance. Computer simulations were conducted using death type (i) and *g*_1_ = *g*_2_ = *g*_3_ = 1. The outcomes of the simulations are presented in Figure 5. When the colony is static (*d*_1_ = *d*_2_ = *d*_3_ = *d*_4_), and the death probabilities relatively low, the 1∶3 long overwhelms 2∶2 short. Colonies adopting the 2∶2 short strategy can outcompete their 1∶3 long counterparts if the death probability of size 1 colonies is sufficiently high. The 1∶3 long strategy is favorable over the 2∶2 short because the latter strategy places a daughter colony only at a nearest neighbor site. Thus, the 2∶2 short strategy cannot search for unoccupied sites when the local colony density is high, and colonies adopting this strategy are prevented from increasing their number. On the other hand, the 1∶3 long strategy disperses a new colony to a random site, releasing it from local density effects. Provided that empty sites exist in the population, colonies adopting the 1∶3 long strategy can sustain population growth. We also conducted simulations in which death probabilities *d*_1_, *d*_2_ and *d*_3_ were variable while *d*_4_ was fixed at 0.2. The outcomes of these simulations are consistent with other results (see Figure S1).

When all of *g*_1_, *g*_2_, and *g*_3_ are less than one, the lattice-structured population displays logistic growth, which favors the 2∶2 short strategy over the 1∶3 long (see Figure S2).

Furthermore, if competition is introduced among all four division/dispersal strategies, colonies adopting either short-distance strategy rapidly become extinct because competition strongly favors long dispersal distance. After just a few iterations, the population comprises colonies adopting the 1∶3 long strategy and the 2∶2 long strategy. Since the 1∶3 and 2∶2 long-dispersing colonies in the lattice-structured population behave identically to their counterparts adopting the 1∶3 and the 2∶2 division strategies in the completely mixed population, the final competition outcomes among all four division/dispersal strategies are those of the 1∶3 and the 2∶2 division strategies in the completely mixed population (see Figure S3).

#### Simulated results in the presence of environmental disturbance

We now introduce two parameters of environmental disturbance, *u* and *q*, defined as the frequency of disturbance and the disturbance scale, respectively [17]. These parameters are independent of the baseline natural death. *q* can be interpreted as the degree of spatial autocorrelation. The probability that a colony of size *i* is removed from a site by environmental disturbance (leaving the previously occupied site empty) is defined as *u_i_* (0.0_≤_ *u_i_*_≤_1.0). Disturbance is assumed independent of colony size and density (*u*_1_ = *u*_2_ = *u*_3_ = *u*_4_ = *u*). A disturbance kills a colony occupying a lattice site with probability *u*. The disturbance then spreads to a nearest neighbor site (selected randomly among eight neighbors) with probability *q* and kills the residential colony (if present). If the site is unoccupied, it is merely disturbed. The disturbance is repeated with probability *q* until halted with probability 1 – *q*. For example, if *u* is low and *q* is high, the lattice contains a few large or clustered disturbed areas. If *u* is high and *q* is low, many small areas are disturbed in the lattice.

**Figure 6:**
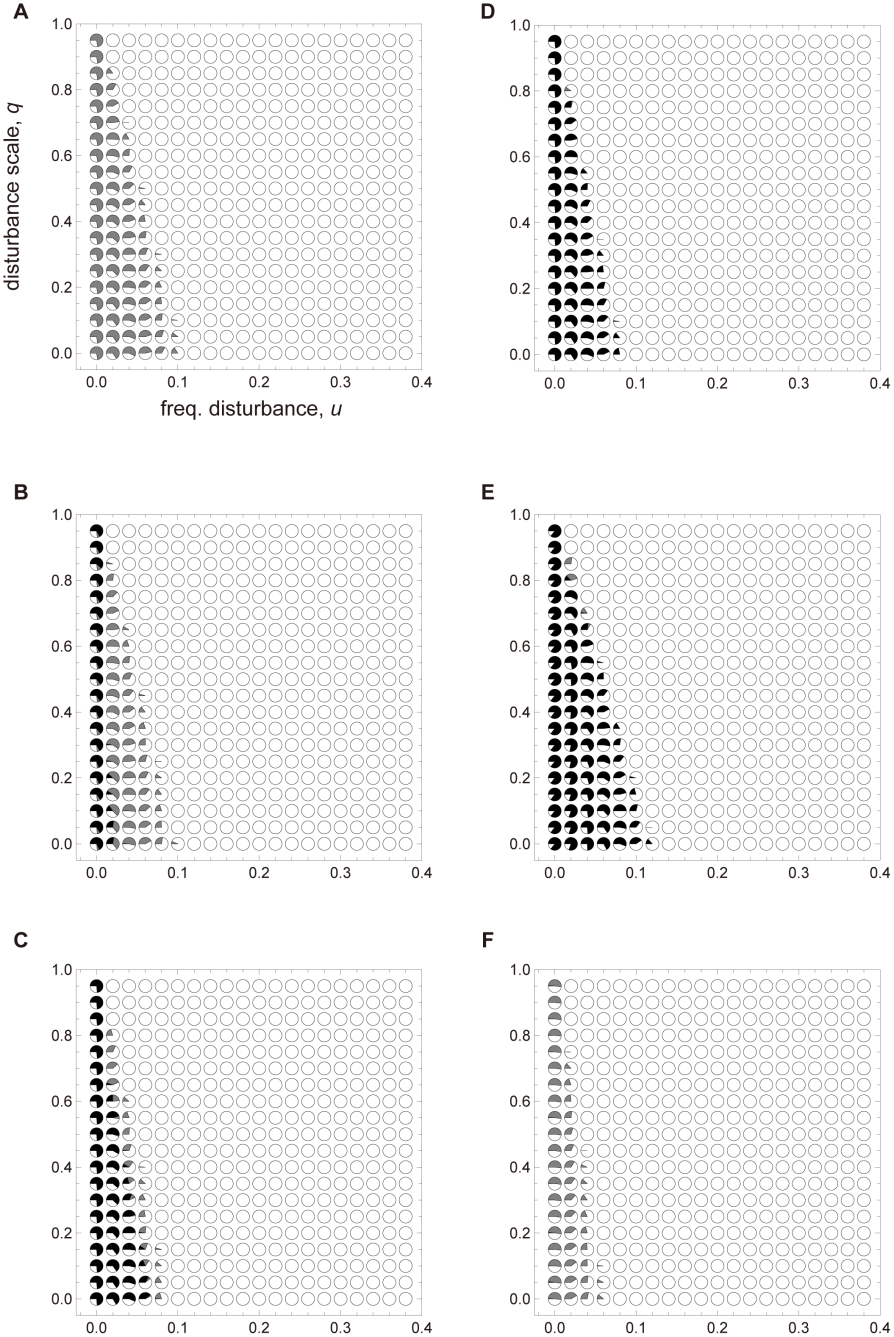
The *u*-*q* graph of colony-based simulations in the two-dimensional lattice-structured population after 5,000 iterations. Details are provided in the caption to Figure 5. The death probability assumes death type (i). (A) *d*_1_ = 0.25 and *d*_2_ = 0.15, (B) *d*_1_ = 0.3 and *d*_2_ = 0.15, (C) *d*_1_ = 0.35 and *d*_2_ = 0.15, (D) *d*_1_ = 0.4 and *d*_2_ = 0.15, (E) *d*_1_ = 0.35 and *d*_2_ = 0.1 and (F) *d*_1_ = 0.35 and *d*_2_ = 0.2. Other parameters are: *g*_1_ = *g*_2_ = *g*_3_ = 1 and *h* = 1.

Figure 6 shows the effect of the disturbance parameters *u* and *q* on the competition between 1∶3 long and 2∶2 short when *g*_1_ = *g*_2_ = *g*_3_ = 1. This simulation assumes death type (i). As demonstrated in Figure 6, low *q* and relatively high *u* confer an advantage to the 2∶2 short strategy when *d*_1_ is higher and the difference between *d*_1_ and *d*_2_ ( = *d*_3_ = *d*_4_) is larger (see figure 6 C, D and E). Increasing *u* and *q* favors the 1∶3 long strategy at certain death probabilities favoring the 2∶2 short strategy in the absence of disturbance. These outcomes are supported by standard dispersal theory. The simulation outcomes are influenced by disturbance when the growth probabilities are less than 1 (see figure S4). According to this figure, the 2∶2 short strategy is advantageous over 1∶3 long when size 2 colonies grow rapidly (high *g*_2_) relative to *g*_1_ (which is fixed; see figure S4 A–C and D–F). Figure S4 A and D, together with Figure S4 B and E, indicate that the 2∶2 short strategy is advantageous when *g*_1_ is low relative to *g*_2_.

## Discussion and Conclusions

We examined the ecological conditions under which budding can be advantageous over migration in colonial organisms. The main conclusions are summarized as follows: (1) when the life history parameters are independent of colony size, budding colonies are overwhelmed by dispersive colonies. (2) When the death probability of the smallest colonies is relatively higher than that of more mature colonies, survival favors the budding strategy. The death function can be modeled as a negative exponential function of the continuous lifespan. (3) Budding wins against dispersal when the growth rates of size 1 and size 3 colonies are lower than that of size 2 colonies. In this scenario, colonies undergo logistic growth, which ensures their survival if they adopt the budding strategy. (4) When a newly-divided dispersing colony does not compete with a settled colony for space, but instead colonizes open spaces, the budding strategy is advantageous over dispersal. This corresponds to the situation in which founding colonies leave their natal site, and seek and settle at a nearby open space.

We then examine the effects of spatial structure and environmental disturbance on the population dynamics. Relative to dispersal, the budding strategy proved less favorable in spatial structured populations than in completely mixed populations. This occurs because long distance dispersal prevents local overcrowding. In the event of environmental disturbance, long distance dispersal confers a survival advantage over budding species because migrating species can escape the risk. However, when the scale of environmental disturbance is small, budding can retain an advantage even under relatively high frequency of environmental disturbance.

Our current paper roughly supports the main results presented in Nakamaru *et al*. (2007). Among the various growth and death functions, logistic colony growth (corresponding to *g*_1_, *g*_3_<*g*_2_) and an exponentially decreasing death function (corresponding to *d*_1_>>*d*_2_, *d*_3_, *d*_4_) favor budding over dispersal. However, the assumptions of our current model differ from those in Nakamaru et al (2007). For example, the previous study assumed continuous colony size, and that each colony obeys logistic growth and an exponentially decreasing death function. Here, to more easily investigate the effect of different growth and death functions on the evolutionary population dynamics, we discretized the colony size. Despite these differences in model assumptions, the results are consistent between the two studies, which strengthens our conclusions.

Budding likely evolves when very small individuals (or colonies) undergo logistic growth and are subject to relatively high mortality. Furthermore, our models suggest that budding is favored when dispersing offspring (colonies) can seek unoccupied spaces in which to settle without inciting conflict. Ant or coral colonies (or colonies of ramets in plants) usually exhibit type III survivorship curves (negative exponential mortality functions) [21]–[23] and logistic growth [21], [24]–[26]. Furthermore, ants are known to seek suitable empty sites when their colony disperses [27], [28]. Thus, these organisms are naturally predisposed to budding. Although our results also suggest that environmental disturbance promotes long-dispersing by its risk hedging effect and discourages budding, the benefits of budding may outweigh the disadvantage of short-distance dispersal in some of the above mentioned organisms (some corals, plants and ants) that reproduce by budding in frequently disturbed environments.

Rigorous empirical testing of our hypothesis is limited by the difficulty of distinguishing the baseline death and growth rates from the changes in those parameters induced by disturbance. However, we consider that our model predictions could be tested on realized average death and growth rates that include both baseline and additional (or reduced) rates caused by disturbance. As already mentioned, life history parameters such as survival and growth functions appear to directly determine which strategy should evolve. If the average death and growth rates of fission-dividing colonial organisms fulfill the predicted conditions favoring the 2∶2 division strategy described in Figure 2 and Eqs. (4) and (6), our models would be supported. The life history of species or closely related species adopting both budding and non-budding tactics must be investigated in empirical studies.

Previous studies on the evolution of body-condition dependency in dispersal have not directly addressed the budding phenomenon [29]–[31]. Instead, these models assume semelparity or an annual lifecycle. Budding is possible only when a parent (or part of the parent's body) survives after reproduction, coexists with the offspring (perennial) and re-reproduces (iteroparity). Therefore, our results are not directly comparable with the results of these studies.

We now discuss the factors that should be incorporated into our current model to render it more realistic. We assumed that a colony reproduces by dividing in two. We also assumed that (not always, however) large offspring disperse over a smaller distance (size-dispersal distance trade off). However, another reproductive constraint has been reported in the literature; the trade-off between size and number of offspring (e.g. [32]). We consider that the size-number trade-off is a sensible proposition when the parental effort or total parental resource that can be allocated to reproduction is constant. For generality in our current study, the total reproductive effort was assumed labile (for instance, the dispersive strategy (3∶1 strategy) invests more resource in survival than budding, given that the dispersed portion is the offspring). We now introduce the 2∶1∶1: division strategy, in which a size 4 colony divides into one size 2 colony and two size 1 colonies that then disperse, and the 1∶1∶1∶1 division strategy, in which a size 4 colony divides into four size 1 colonies. We constrained the reproduction effort to be constant (mimicking a size–number trade off) in the competition between 2∶1∶1 vs. 2∶2 strategies. However, biological strategies might be more precisely described by 2∶1∶1 and 1∶1∶1∶1 strategies; for example; plants may release many seeds and ant colonies disperse many winged queens simultaneously. We have partially tackled this situation. In well-mixed populations, the local stable conditions of the 1∶1∶1∶1 and the 2∶1∶1 division strategies are 4*p*_1_ *p*_2_ *p*_3_ > *p*_4_ + (1−*p*_4_)/*h* and *p*_2_ *p*_3_(1+2*p*_1_) > *p*_4_ + (1−*p*_4_)/*h*, respectively (method presented in the Appendices). The left hand side of both inequalities specifies the probability that dividing colonies mature to size 4. This result implies that (i) budding wins against the other three strategies when the survival probability of size 1 colonies (*p*_1_) is very small, and (ii) if the converse is true (*p*_1_ is high), the 1∶1∶1∶1 division strategy may be favored. The latter condition might simulate the evolution of semelparity. However, the strongest strategy when members of the population adopt all four strategies has yet to be elucidated.Furthermore, one must consider relaxing the assumption on the timing of reproduction. Size 4 colonies only reproduce in the current model. If we assume that size 2 and 3 colonies can reproduce, we can analyze the conditions under which annual or perennial life cycles evolve (regarding the reproductive strategy of size 2 colonies as annual semelparity).

The current model does not include colony size effects in disturbance. For instance, if smaller colonies are more vulnerable to environmental disturbance than larger ones, the budding might be more advantageous over dispersal than is predicted in this paper. Finally, our study considers neither explicit competition among individuals within each colony nor direct interference induced by competition between neighboring colonies. In future work, we will incorporate these factors into a more realistic model.

Budding has been considered to play a key role in evolutionary ecology. Theoretically, budding increases the genetic variance among groups and/or the genetic relatedness within groups, thus promoting the evolution of group-advantageous traits such as altruism and female-biased sex allocation [33]–[35]. On the other hand, restricted migration (which frequently accompanies fission) intensifies the local competition among related individuals and hinders the evolution of altruism, despite the apparent enhancement of genetic relatedness of interactants [36]–[38]. However, recent theoretical and empirical studies suggest that budding can solve this “viscous population dilemma” and enable the evolution of altruism, because budding increases the number of more related individuals in the background local population [7], [8], [39]–[45]. All of the above-mentioned theoretical studies focus on the effect of budding on evolved social traits. Thus, budding is assumed as the given condition, without considering the environments in which budding evolves. The latter problem is the focus of our current paper. On the other hand, the ecological life history models introduced here neglect issues such as relatedness and intra-colonial conflicts. Therefore, our current study is complementary to existing studies. In future studies, we hope to incorporate relatedness and intra-colonial conflicts into the model scheme, to better understand the ecological drivers of coevolving life history and social traits.

## Supporting Information

Figure S1 **Effect of life history parameters on competition between the two strategies in the two-dimensional lattice.** Black spheres indicate that the 2∶2 short strategy overcomes the 1∶3 long strategy, while gray spheres indicate the opposite. Where no spheres are presented, colonies have become extinct. The volume of each sphere denotes the average density of the strategy after 1,000 iterations. This graph shows the effect of imposing the death probabilities *d*_1_, *d*_2_, *d*_3_. The parameters are *d*_4_ = 0.2, *g*_1_ = *g*_2_ = *g*_3_ = 1, *h* = 1. Initial densities are *z*_0_ = 0.6 and *x*_1_ = *x*_2_ = *x*_3_ = *x*_4_ = *y*_1_ = *y*_2_ = *y*_3_ = *y*_4_ = 0.05.
(TIF)

Figure S2 **Effect of life history parameters on competition between the two strategies in the two-dimensional lattice.** Black spheres indicate that the 2∶2 short strategy overcomes the 1∶3 long strategy, while gray spheres indicate the opposite. Where no spheres are presented, the colonies have become extinct. The volume of each sphere denotes the average density of the strategy after 6,250 iterations. This graph illustrates the effect of growth probabilities *g*_1_, *g*_2_, and *g*_3_. Other parameters are: *d*_1_ = 0.35, *d*_2_ = *d*_3_ = *d*_4_ = 0.15. Initial densities are *z*_0_ = 0.6 and *x*_1_ = *x*_2_ = *x*_3_ = *x*_4_ = *y*_1_ = *y*_2_ = *y*_3_ = *y*_4_ = 0.05.
(TIF)

Figure S3 **Competition among four possible strategies in a structured population residing on a two dimensional lattice, after 10,000 iterations.** The parameters are *g*_2_ = *g*_3_, *d*_1_ = 0.35, *d*_2_ = *d*_3_ = *d*_4_ = 0.15 and *h* = 1. The initial population density of each strategy is the same and the initial density of vacant sites is 0.6.
(TIF)

Figure S4 **The** ***u*** **-** ***q*** **graph of colony-based simulations in the two-dimensional lattice structured population after 6,250 iterations.** Details are provided in the caption to Figure 5. (A) *g*_1_ = 0.8 and *g*_2_ = 0.8, (B) *g*_1_ = 0.8 and *g*_2_ = 0.6, (C) *g*_1_ = 0.8 and *g*_2_ = 0.4, (D) *g*_1_ = 0.6 and *g*_2_ = 0.8, (E) *g*_1_ = 0.6 and *g*_2_ = 0.6 and (F) *g*_1_ = 0.6 and *g*_2_ = 0.4. Other parameters are: *d*_1_ = 0.35, *d*_2_ = *d*_3_ = *d*_4_ = 0.15, *h* = 1 and *g*_3_ = 0.9. Initial density of each size of each strategy is identical, and the initial density of vacant sites is 0.6.
(TIF)

Appendix S1 **Corollary of Jury's criterion.**
(DOC)

Appendix S2 **Local stability analysis of positive equilibrium on the 2**∶**2 division strategy.**
(DOC)

Appendix S3 **Local stability analysis of positive equilibrium on the 1**∶**3 division strategy.**
(DOC)

Appendix S4 **Local stability analysis in competitive system between two strategies.**
(DOC)

## References

[1] Clobert J, Danchin E, Dhonde AA, Nichols JD (2001) Dispersal. Oxford: Oxford University Press.

[2] Levin SA, Muller-Landau HC, Nathan R, Chave J (2003) The ecology and evolution of seed dispersal: A theoretical perspective. Annu Rev Ecol Syst 34: 575–604.

[3] Clobert J, Baguette M, Benton TG, Bullock JM (2012) Dispersal ecology and evolution. Oxford: Oxford University Press.

[4] Wynee-Edwards V (1962) Animal dispersion in relation to social behaviour. New York: Hafaer.

[5] Williams GC (1966) Adaptation and natural selection. Princeton: Princeton University Press.

[6] Ronce O, Olivieri I, Clobert J, Danchin E (2001) Perspectives on the study of dispersal evolution. In: Clobert J, Danchin E, Dhonde AA, Nichols JD, editors. Dispersal. Oxford: Oxford University Press. pp. 341–357.

[7] Gardner A, West SA (2006) Demography, altruism, and the benefits of budding. J Evol Biol 19: 1707–1716. 16911000 10.1111/j.1420-9101.2006.01104.x

[8] Kummerli R, Gardner A, West SA, Griffin AS (2009) Limited dispersal, budding dispersal, and cooperation: an experimental study. Evolution 63: 939–949. 19154373 10.1111/j.1558-5646.2008.00548.x

[9] Gardner A, Arce A, Alpedrinha J (2009) Budding dispersal and the sex ratio. Journal of Evolutionary Biology 22: 1036–1045. 21462403 10.1111/j.1420-9101.2009.01719.x

[10] Liu J, Dong M, Miao SL, Li ZY, Song MH, et al (2006) Invasive alien plants in China: role of clonality and geographical origin. Biol Invasions 8: 1461–1470.

[11] Honnay O, Jacquemyn H (2010) Clonal plants: beyond the patterns—ecological and evolutionary dynamics of asexual reproduction. Evol Ecol 24: 1393–1397.

[12] Coffroth MA, Lasker HR (1998) Population structure of a clonal gorgonian coral: The interplay between clonal reproduction and disturbance. Evolution 52: 379–393. 28568334 10.1111/j.1558-5646.1998.tb01639.x

[13] Passera L (1994) Characteristics of tramp species. In: William DF, editor. Exotic ants: biology, impact, and control of introduced species. Colorado: Westview Press. pp. 23–43.

[14] Holway DA, Lach L, Suarez AV, Tsutsui ND, Case TJ (2002) The causes and consequences of ant invasions. Annu Rev Ecol Syst 33: 181–233.

[15] Carpintero S, Reyes-Lopez J, de Reyna LA (2003) Impact of human dwelling on the distribution of the exotic Argentine ant: a case study in the Doñana National Park. Biol Conserv 115: 279–289.

[16] Tsuji K (2010) Unicolonial ants: loss of colony identity. In: Breed M, Moore J, editors. Encyclopedia of Animal Behavior. Oxford: Academic Press. pp. 469–473.

[17] Nakamaru M, Beppu Y, Tsuji K (2007) Does disturbance favor dispersal? An analysis of ant migration using the colony-based lattice model. J Theor Biol 248: 288–300. 17583750 10.1016/j.jtbi.2007.05.012

[18] Caswell H (2000) Matrix population models 2nd edition. Sunderland: Sinauer Associates, Inc., Publishers.

[19] Stearns SC (1992) The Evolution of Life Histories. Oxford: Oxford University Press.

[20] Gardner A, Grafen A (2009) Capturing the superorganism: a formal theory of group adaptation. J Evol Biol 22: 659–671. 19210588 10.1111/j.1420-9101.2008.01681.x

[21] Hölldobler B, Wilson EO (1990) The ants. Cambridge: Belknap Press of Harvard University Press.

[22] Babcock RC (1991) Comparative demography of three species of scleractinian corals using age- and size-dependent classifications. Ecol Monogr 61: 225–244.

[23] Harper JL (1977) Population Biology of Plants. Waltham: Academic Press.

[24] Yamaguchi M (1983) Growth data analysis in the reef-building coral {Pocillopora} {damicornis} (Linnaeus). Galaxea 2: 21–27.

[25] van Woesik R, Sakai K, Ganase A, Loya Y (2011) Revisiting the winners and the losers a decade after coral bleaching. Mar Ecol Prog Ser 434: 67–76.

[26] Suzuki JI, Hucthings MJ (1997) Interactions between shoots in clonal plants and the effects of stored resources on the structure of shoot population. In: de Kroon H, van Groenendael J, editors. The Ecology and Evolution of Clonal Plants. Leiden: Backhuys Publishers.

[27] Stroeymeyt N, Giurfa M, Franks NR (2010) Improving decision speed, accuracy and group cohesion through early information gathering in house-hunting ants. PLoS One 5: e13059. 20927374 10.1371/journal.pone.0013059 PMC2947506

[28] Cronin AL (2012) Consensus decision making in the ant Myrmecina nipponica: house-hunters combine pheromone trails with quorum responses. Anim Behav 84: 1243–1251.

[29] Gyllenberg M, Kisdi E, Utz M (2008) Evolution of condition-dependent dispersal under kin competition. J Math Biol 57: 285–307. 18259755 10.1007/s00285-008-0158-2

[30] Gyllenberg M, Kisdi E, Utz M (2011) Body condition dependent dispersal in a heterogeneous environment. Theor Popul Biol 79: 139–154. 21426910 10.1016/j.tpb.2011.02.004

[31] Gyllenberg M, Kisdi E, Utz M (2011) Variability within families and the evolution of body-condition-dependent dispersal. J Biol Dyn 5: 191–211. 22873439 10.1080/17513758.2010.519403

[32] Smith CC, Fretwell SD (1974) The optimal balance between size and number of offspring. Am Nat 108: 499–506.

[33] Slatkin M, Wade MJ (1978) Group selection on a quantitative character. Proc Natl Acad Sci U S A 75: 3531–3534. 16592546 10.1073/pnas.75.7.3531 PMC392812

[34] Goodnight CJ, Stevens L (1997) Experimental studies of group selection: what do they tell us about group selection in nature? Am Nat 150: S59–S79. 18811313 10.1086/286050

[35] Ono S, Misawa K, Tsuji K (2003) Effect of Group Selection on the Evolution of Altruistic Behavior. J Theor Biol 220: 55–66. 12453450 10.1006/jtbi.2003.3144

[36] Wilson DS, Pollock GB, Dugatkin LA (1992) Can sltruism evolve in purely viscous populations? Evol Ecol 6: 331–341.

[37] Taylor PD (1992) Altruism in viscous populations - an inclusive fitness model. Evol Ecol 6: 352–356.

[38] Taylor PD (1992) Inclusive fitness in a homogeneous environment. Proc Biol Sci 249: 299–302.

[39] Sharp SP, Simeoni M, Hatchwell BJ (2008) Dispersal of sibling coalitions promotes helping among immigrants in a cooperatively breeding bird. Proc Biol Sci 275: 2125–2130. 18522914 10.1098/rspb.2008.0398 PMC2603207

[40] Slatkin M (1977) Gene flow and genetic drift in a species subject to frequent local extinctions. Theor Popul Biol 12: 253–262. 601717 10.1016/0040-5809(77)90045-4

[41] Goodnight KT (1992) The effect of stochastic variation on kin selection in a budding-viscous population. Am Nat 140: 1028–1040. 19426032 10.1086/285454

[42] Lehmann L, Perrin N, Rpusset F (2006) Population demography and the evolution of helping behaviors. Evolution 60: 1137–1151. 16892965

[43] Bradley BJ, Doran-Sheehy DM, Vigilant L (2007) Potential for female kin associations in wild western gorillas despite female dispersal. Proc Biol Sci 274: 2179–2185. 17609183 10.1098/rspb.2007.0407 PMC2706186

[44] Heinsohn R, Dunn P, Leggs S, Double M (2000) Coalitions of relatives and reproductive skew in cooperatively breeding white-winged choughs. Proc Biol Sci 267: 243–249. 10714878 10.1098/rspb.2000.0993 PMC1690523

[45] Williams DA, Rabenold KN (2004) Male-biased dispersal, female philopatry, and routes to fitness in a social corvid. J Anim Ecol 74: 150–159.

