# Supplementary figures and images for: Ecological Conditions Favoring Budding in Colonial Organisms under Environmental Disturbance
Source: PLoS One. 2014 Mar 12;9(3):e91210. doi: 10.1371/journal.pone.0091210 (PMC3951312; doi:10.1371/journal.pone.0091210)

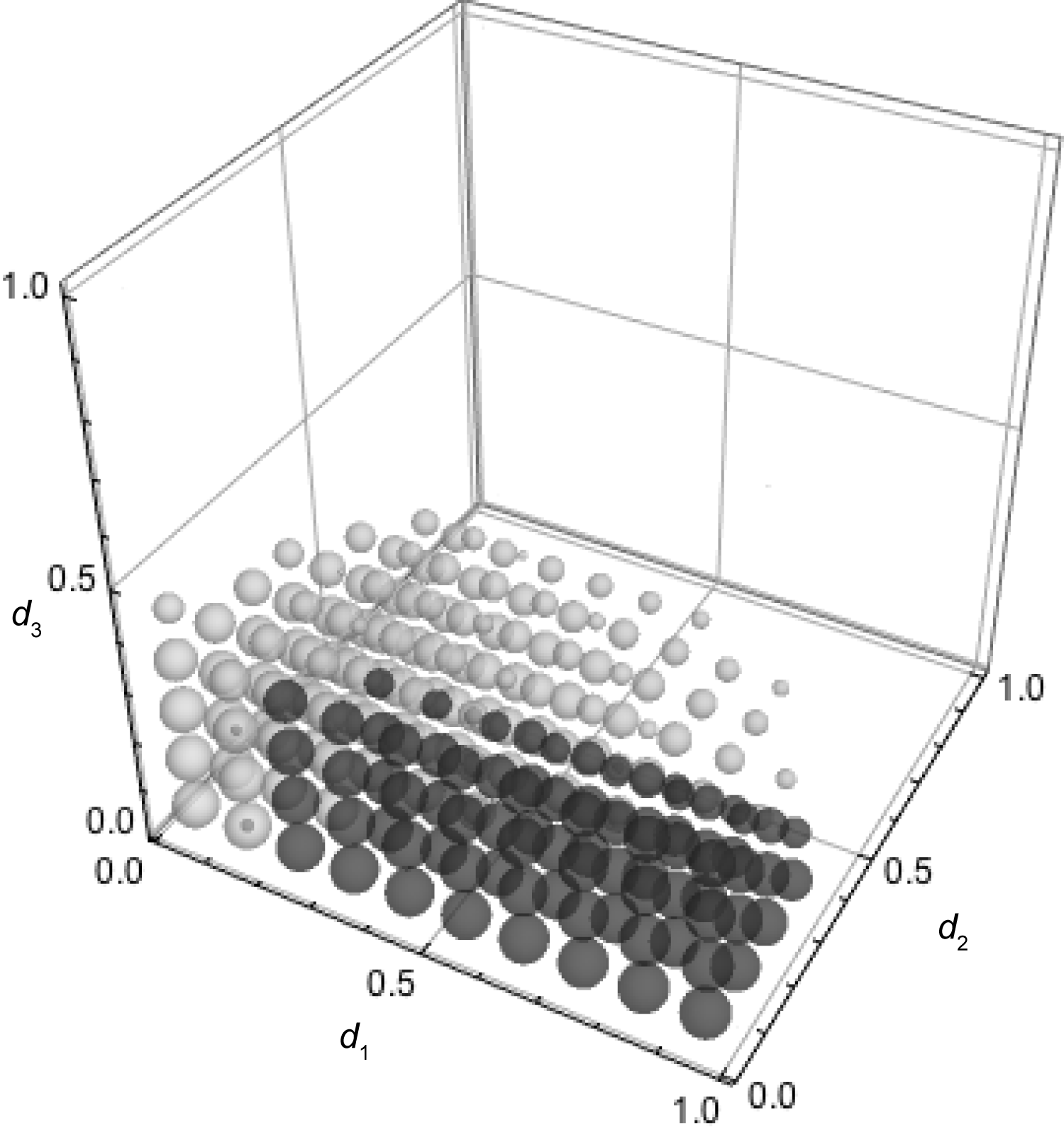

Supplement: Figure S1 — Effect of life history parameters on competition between the two strategies in the two-dimensional lattice. Black spheres indicate that the 2∶2 short strategy overcomes the 1∶3 long strategy, while gray spheres indicate the opposite. Where no spheres are presented, colonies have become extinct. The volume of each sphere denotes the average density of the strategy after 1,000 iterations. This graph shows the effect of imposing the death probabilities d 1, d 2, d 3. The parameters are d 4 = 0.2, g 1 = g 2 = g 3 = 1, h = 1. Initial densities are z 0 = 0.6 and x 1 = x 2 = x 3 = x 4 = y 1 = y 2 = y 3 = y 4 = 0.05. (TIF) [file pone.0091210.s001.tif]

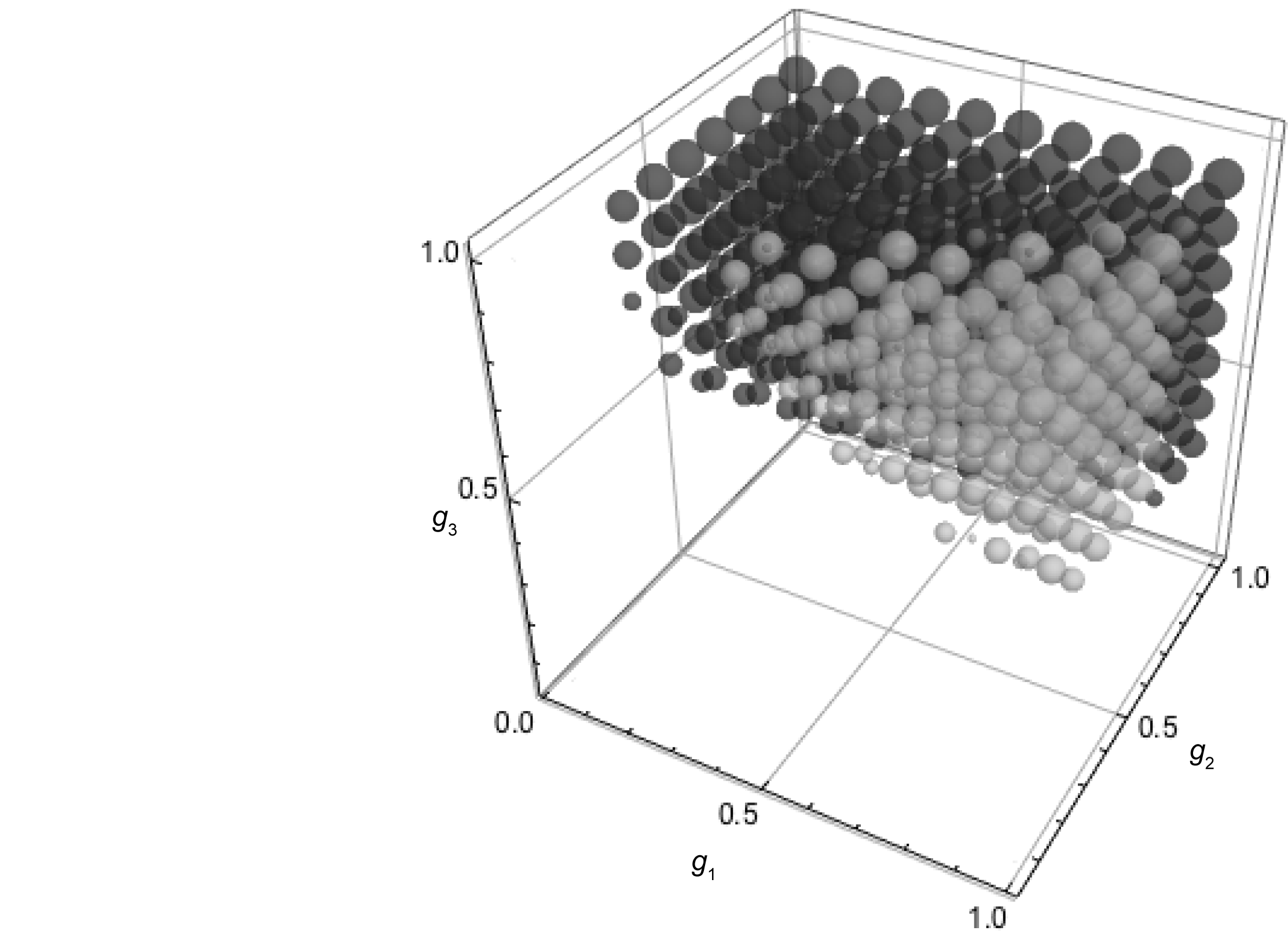

Supplement: Figure S2 — Effect of life history parameters on competition between the two strategies in the two-dimensional lattice. Black spheres indicate that the 2∶2 short strategy overcomes the 1∶3 long strategy, while gray spheres indicate the opposite. Where no spheres are presented, the colonies have become extinct. The volume of each sphere denotes the average density of the strategy after 6,250 iterations. This graph illustrates the effect of growth probabilities g 1, g 2, and g 3. Other parameters are: d 1 = 0.35, d 2 = d 3 = d 4 = 0.15. Initial densities are z 0 = 0.6 and x 1 = x 2 = x 3 = x 4 = y 1 = y 2 = y 3 = y 4 = 0.05. (TIF) [file pone.0091210.s002.tif]

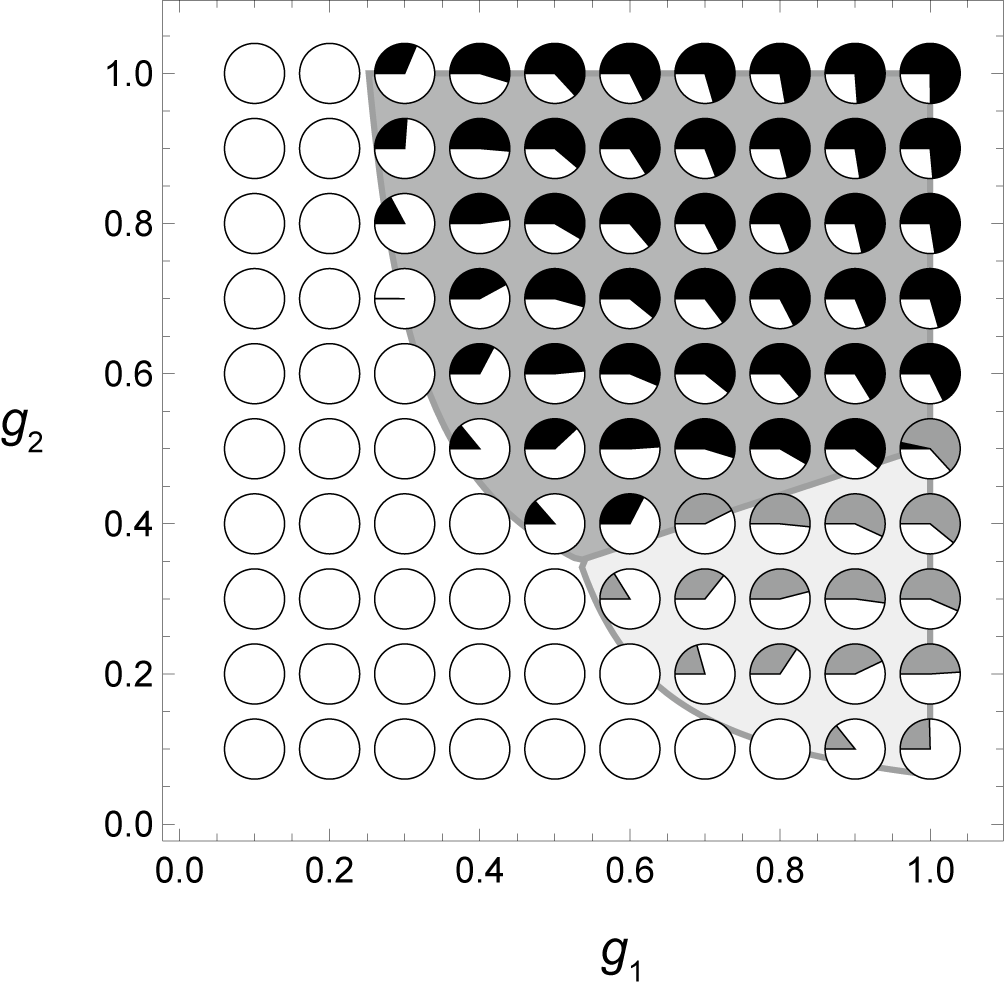

Supplement: Figure S3 — Competition among four possible strategies in a structured population residing on a two dimensional lattice, after 10,000 iterations. The parameters are g 2 = g 3, d 1 = 0.35, d 2 = d 3 = d 4 = 0.15 and h = 1. The initial population density of each strategy is the same and the initial density of vacant sites is 0.6. (TIF) [file pone.0091210.s003.tif]

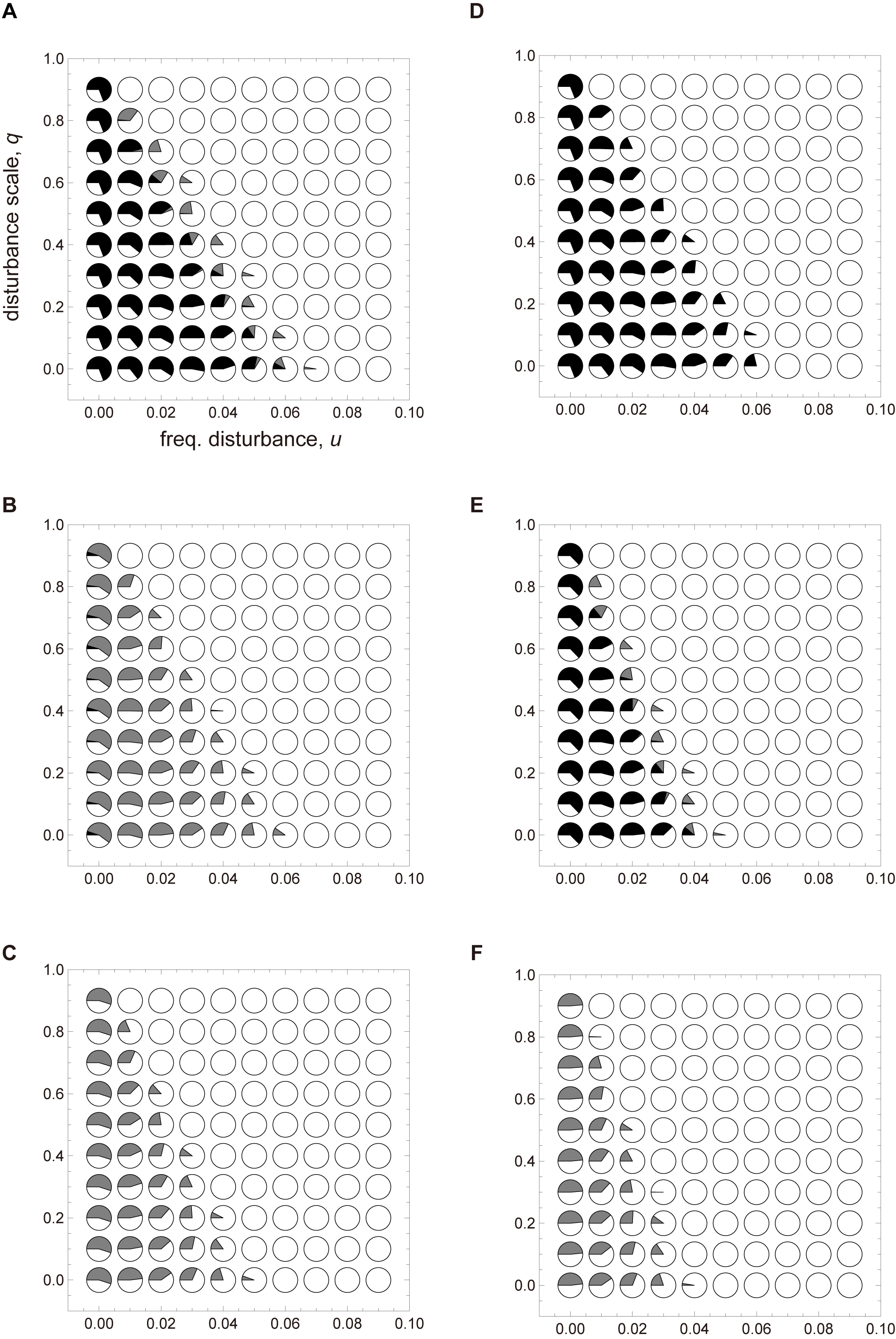

Supplement: Figure S4 — The u - q graph of colony-based simulations in the two-dimensional lattice structured population after 6,250 iterations. Details are provided in the caption to Figure 5. (A) g 1 = 0.8 and g 2 = 0.8, (B) g 1 = 0.8 and g 2 = 0.6, (C) g 1 = 0.8 and g 2 = 0.4, (D) g 1 = 0.6 and g 2 = 0.8, (E) g 1 = 0.6 and g 2 = 0.6 and (F) g 1 = 0.6 and g 2 = 0.4. Other parameters are: d 1 = 0.35, d 2 = d 3 = d 4 = 0.15, h = 1 and g 3 = 0.9. Initial density of each size of each strategy is identical, and the initial density of vacant sites is 0.6. (TIF) [file pone.0091210.s004.tif]
