# Supplementary material for: Ecological Conditions Favoring Budding in Colonial Organisms under Environmental Disturbance
Source: PLoS One. 2014 Mar 12;9(3):e91210. doi: 10.1371/journal.pone.0091210 (PMC3951312; doi:10.1371/journal.pone.0091210)
Supplement: Appendix S1 — Corollary of Jury's criterion. (DOC) [file pone.0091210.s005.doc]

Supporting Information for " Ecological conditions favoring budding in colonial organisms under environmental disturbance," Mayuko Nakamaru, Takenori Takada, Akiko Ohtsuki, Sayaki, U. Suzuki, Kanan Miura, Kazuki Tsuji

Appendix S1: Corollary of Jury’s criterion

In this appendix, we will prove two corollaries on Jury’s criterion, which is the necessary and sufficient condition of the local stability analysis at equilibrium in difference equations, to apply them into the analysis in Appendix S2 and S3. Consider a dynamical difference equation:

$\mathbf{z}(t+1)=\mathbf{P}\left(\mathbf{z}(t)\right)\mathbf{z}(t)$ , (A1)

where **z**(*t*) and **P**(**z**(*t*)) are a state vector at time *t* and **z**(*t*)-dependent transition matrix, respectively. Denoting **w**(*t*)= **z**(*t*)- **z*** and linearizing Eq. (A1) at an equilibrium **z***, we obtain

$\mathbf{w}(t+1)=\mathbf{J}\mathbf{w}(t)$ , (A2)

where **J** is a Jacobian matrix. Jury’s criterion is the necessary and sufficient condition that all the eigenvalues of **J** are within a unit circle on complex plane (Jury 1974; Jury 1982).

According to Jury’s criterion, when a vector **z**(*t*) is three-dimensional, the characteristic equation of the Jacobian and the condition can be described as

, (A3)

and

*f*(1) > 0 1 + *a*_2_ + *a*_1_ + *a*_0_ > 0, (A4-1)

(−1)^3^*f*(−1) > 0 1 − *a*_2_ + *a*_1_ − *a*_0_ > 0, (A4-2)

$\left|a_{0}\right|<1$, (A4-3)

. (A4-4)

The Jury’s criterion inequalities, ineq. (A4), can lead to the following corollary.

Corollary 1. In three-dimensional case, if $a_{i}<0(i=0,1,2)$, then the condition that all the eigenvalues satisfy $\left|\lambda_{i}\right|<1$ is $1+a_{2}+a_{1}+a_{0}>0$.

Proof. i) Ineq. (A4-2) leads to $1+a_{2}+a_{1}+a_{0}>2\left(a_{2}+a_{0}\right)$. Because$a_{i}<0$, the right-hand side of the inequality is negative. Therefore, ineqs. (A4-1) and (A4-2) is equivalent with ineq. (A4-1).

ii) Because $a_{0}<0$, $\left|a_{0}\right|<1$ 1 + *a*_0_ > 0 1 + *a*_2_ + *a*_1_ + *a*_0_ > *a*_2_ + *a*_1_. The right-hand side of the inequality is negative under the assumption $a_{i}<0$. Therefore, ineqs. (A4-1) and (A4-3) are equivalent with ineq. (A4-1).

iii) Similarly, $1-a_{0}^{2}>a_{0}a_{2}-a_{1}$ $1+a_{0}>\frac{a_{0}}{1-a_{0}}a_{2}-\frac{1}{1-a_{0}}a_{1}$

since $a_{i}<0$ and 1 + *a*_0_ > 0. Therefore, 1 + *a*_2_ + *a*_1_ + *a*_0_ > $\frac{1}{1-a_{0}}a_{2}-\frac{a_{0}}{1-a_{0}}a_{1}$. Because the right-hand side of the inequality is negative, ineqs. (A4-1) and (A4-4) are equivalent with (A4-1). Therefore, four inequalities from (A4-1) to (A4-4) are summarized to (A4-1). It should be noted that the same conclusion is derived when $a_{0}<0,a_{1}=0,a_{2}<0$. <Q.E.D>

Similarly, in the case of four dimensions, characteristic equation of the Jacobian and the condition can be described as

$f\left(\lambda\right)=\lambda^{4}+a_{3}\lambda^{3}+a_{2}\lambda^{2}+a_{1}\lambda+a_{0}=0$ , (A5)

and

*f*(1) > 0 1 + *a*_3_ + *a*_2_ + *a*_1_ + *a*_0_ > 0, (A6-1)

(−1)^4^*f*(−1) > 0 1 − *a*_3_ + *a*_2_ − *a*_1_ + *a*_0_ > 0, (A6-2)

$\left|a_{0}\right|<1$ (A6-3)

, (A6-4)

. (A6-5)

The Jury’s criterion inequalities, (A6), leads to the following corollary.

Corollary 2. In four-dimensional case, if $a_{i}<0(i=0,\cdots3)$, then the condition that all the eigenvalues satisfy $\left|\lambda_{i}\right|<1$ is $1+a_{3}+a_{2}+a_{1}+a_{0}>0$.

<Proof> Following the proof of corollary 1, it is proved that ineqs. (A6-1), (A6-2), (A6-3) and (A6-4) is summarized to (A6-1). We here only prove that ineqs. (A6-1) and (A6-5) are equivalent to ineq. (A6-5).

First of all, we will prove $1-a_{0}^{2}>a_{0}a_{3}-a_{1}$.

$$1-a_{0}^{2}-\left(a_{0}a_{3}-a_{1}\right)$$

= $\left(1-a_{0}\right)\left\{1+a_{0}-\frac{a_{0}}{1-a_{0}}a_{3}+\frac{1}{1-a_{0}}a_{1}\right\}$ , (A7)

= $\left(1-a_{0}\right)\left\{1+a_{3}+a_{2}+a_{1}+a_{0}-a_{2}-\frac{1}{1-a_{0}}a_{3}+\frac{a_{0}}{1-a_{0}}a_{1}\right\}>0$

when ineq. (A6-1) holds because $a_{i}<0$. Therefore, the left-hand side of (A6-5) can be rewritten as:

$\left|\left(a\right._{0}^{2}-\left.1\right)^{2}-\left(a_{0}a_{3}-a_{1}\right)^{2}\right|=\left(1-a\right._{0}^{2})^{2}-\left(a_{0}a_{3}-a_{1}\right)^{2}$ . (A8)

The right-hand side of (A6-5) can be rewritten as:

$\left|\left(a\right._{0}^{2}-\left.1\right)a_{2}\left(a_{0}-1\right)-\left(a_{0}a_{1}-a_{3}\right)\left(a_{0}a_{3}-a_{1}\right)\right|=\left(1-a_{0}^{2}\right)a_{2}\left(a_{0}-1\right)+\left(a_{0}a_{1}-a_{3}\right)\left(a_{0}a_{3}-a_{1}\right)$ , (A9)

because $a_{i}<0$ and$1+a_{0}>0$.

Subtracting (A9) from (A8), we can obtain;

$\begin{matrix} \left(1-a\right._{0}^{2})^{2}-\left(a_{0}a_{3}-a_{1}\right)^{2}-\left(1-a_{0}^{2}\right)a_{2}\left(a_{0}-1\right)+\left(a_{0}a_{1}-a_{3}\right)\left(a_{0}a_{3}-a_{1}\right) \\ =\left(1\right.-\left.a_{0}^{2}\right)\left\{\left(1\right.\right.-\left.a_{0}^{2}\right)-a_{2}\left.\left(a_{0}-1\right)\right\}-\left(a_{0}\right.a_{3}-\left.a_{1}\right)\left\{\left(a_{0}a_{3}\right.\right.-\left.a_{1}\right)+a_{0}a_{1}-\left.a_{3}\right\} \\ =\left(1\right.-\left.a_{0}^{2}\right)(1-a_{0})(1+a_{0}+a_{2})+(1-a_{0})\left(a_{0}a_{3}\right.-\left.a_{1}\right)\left(a\right._{1}+\left.a_{3}\right) \\ =\left(1\right.-\left.a_{0}\right)\left\{\left(1\right.\right.-\left.a_{0}^{2}\right)\left(1+\left.a_{3}+a_{2}+a_{1}+a_{0}\right)-\right.\left(a_{1}+a_{3}\right)\left.\left(1-a_{0}^{2}-\left(a_{0}\right.a_{3}\right.\left.\left.-a_{1}\right)\right)\right\} \end{matrix}$ ,

which is positive when (A6-1) holds because , and (A7). Therefore, five inequalities from (A6-1) to (A6-5) are summarized to (A6-1). It should be noted that the same conclusion is derived when $a_{0}<0,a_{1}=0,a_{2}<0\text{ and }a_{3}<0$. <Q.E.D>

Literature cited

Jury, E. I. 1974, Inners and Stability of Dynamic Systems. New York, Wiley.

Jury, E. I. 1982, Inners and Stability of Dynamic Systems. Florida, Krieger Publishing Company.
