# Supplementary material for: Ecological Conditions Favoring Budding in Colonial Organisms under Environmental Disturbance
Source: PLoS One. 2014 Mar 12;9(3):e91210. doi: 10.1371/journal.pone.0091210 (PMC3951312; doi:10.1371/journal.pone.0091210)
Supplement: Appendix S2 — Local stability analysis of positive equilibrium on the 2∶2 division strategy. (DOC) [file pone.0091210.s006.doc]

Supporting Information for " Ecological conditions favoring budding in colonial organisms under environmental disturbance," Mayuko Nakamaru, Takenori Takada, Akiko Ohtsuki, Sayaki, U. Suzuki, Kanan Miura, Kazuki Tsuji

Appendix S2: Local stability analysis of positive equilibrium on the 2:2 division strategy

From eq. (1) in the main text, the dynamics of the 2:2 division strategy follows the difference equation as:

$\mathbf{x}(t+1)=\mathbf{A}\left(\mathbf{x}(t)\right)\mathbf{x}(t)$ , (1)

or

.

Note that we assume

$p_{1}\leq p_{2}\leq p_{3}\leq p_{4}$ , (B1)

because the survival rate of bigger colony is usually larger than that of smaller one and$0<h\leq1$. There are two equilibrium points that satisfy

$\mathbf{x}*=\mathbf{A}*\left(\mathbf{x}*\right)\mathbf{x}*$ , (B2)

in the dynamics of Eq. (1). They are $\mathbf{x}*=(1,0,0,0,0)^{\text{T}}$ and

$\mathbf{x}*=\left(\frac{A}{h},0,\frac{2A(h-A)}{h(1+2A+2p_{2}A)},\frac{2p_{2}A(h-A)}{h(1+2A+2p_{2}A)},\frac{h-A}{h(1+2A+2p_{2}A)}\right)^{\text{T}}$, (B3)

where $A=\frac{1-p_{4}}{2p_{2}p_{3}-p_{4}}$ and T represents transposed form of a vector. *A* and *h-A* should be positive for the existence of non-trivial equilibrium and

$2p_{2}p_{3}>p_{4}+\frac{1-p_{4}}{h}$ . (B4)

The Jacobian matrix of frequent-dependent matrix, $S\left(\mathbf{x}(t)\right)$can be obtained by using the following formula:

$J=S*+\left(\begin{matrix} & \frac{dS}{dx_{k}}\mathbf{x}* \end{matrix}\right)$ , (B5)

at equilibrium, where $\frac{dS}{dx_{k}}\mathbf{x}$ represents the *k*-th column of a matrix (Caswell 2000: Chapter 16). Therefore, the Jacobian matrix at non-trivial equilibrium is

$$\mathbf{J}=\left(\begin{matrix} 1-hx_{4}^{*}-h(1-p_{4})x_{4}^{*} & (1-p_{1}) & (1-p_{2}) & (1-p_{3}) & (1-p_{4})(1-hx_{0}^{*})-hx_{0}^{*} \\ 0 & 0 & 0 & 0 & 0 \\ 2hx_{4}^{*} & p_{1} & 0 & 0 & 2hx_{0}^{*} \\ 0 & 0 & p_{2} & 0 & 0 \\ -hp_{4}x_{4}^{*} & 0 & 0 & p_{3} & p_{4}(1-hx_{0}^{*}) \end{matrix}\right)$$

and the characteristic equation is

$DetS=\left|\begin{matrix} 1-hx_{4}^{*}-h(1-p_{4})x_{4}^{*}-\lambda& (1-p_{1}) & (1-p_{2}) & (1-p_{3}) & (1-p_{4})(1-hx_{0}^{*})-hx_{0}^{*} \\ 0 & -\lambda& 0 & 0 & 0 \\ 2hx_{4}^{*} & p_{1} & -\lambda& 0 & 2hx_{0}^{*} \\ 0 & 0 & p_{2} & -\lambda& 0 \\ -hp_{4}x_{4}^{*} & 0 & 0 & p_{3} & p_{4}(1-hx_{0}^{*})-\lambda\end{matrix}\right|=0$.

i) The stability of the trivial equilibrium: $(1,0,0,0,0)^{\text{T}}$

The characteristic equation at the trivial equilibrium is

$$DetS_{0}=\left|\begin{matrix} 1-\lambda& (1-p_{1}) & (1-p_{2}) & (1-p_{3}) & (1-p_{4})(1-h)-h \\ 0 & -\lambda& 0 & 0 & 0 \\ 0 & p_{1} & -\lambda& 0 & 2h \\ 0 & 0 & p_{2} & -\lambda& 0 \\ 0 & 0 & 0 & p_{3} & p_{4}(1-h)-\lambda\end{matrix}\right|$$

$=(1-\lambda)\left|\begin{matrix} -\lambda& 0 & 0 & 0 \\ p_{1} & -\lambda& 0 & 2h \\ 0 & p_{2} & -\lambda& 0 \\ 0 & 0 & p_{3} & p_{4}(1-h)-\lambda\end{matrix}\right|$ (B6)

$=(1-\lambda)(-\lambda)\left|\begin{matrix} -\lambda& 0 & 2h \\ p_{2} & -\lambda& 0 \\ 0 & p_{3} & p_{4}(1-h)-\lambda\end{matrix}\right|=(1-\lambda)(-\lambda)f(\lambda)=0$.

It should be noted that the Jacobian at equilibrium has the eigenvalues, 0 and 1, because there occurs no colony with size 1 and because the sum of *x_i_* is always equal to one, respectively. Therefore, we should examine the other eigenvalues of

$f(\lambda)=\lambda^{3}+a_{2}\lambda^{2}+a_{1}\lambda+a_{0}=\lambda^{3}+\{-p_{4}(1-h)\}\lambda^{2}-2hp_{2}p_{3}$,

where $a_{2}=-p_{4}(1-h)$, $a_{1}=0$ and $a_{0}=-2hp_{2}p_{3}$. Since $a_{0},a_{2}<0$ and , Corollary 1 in Appendix S1 can be applied to evaluate the eigenvalues and the necessary and sufficient condition of local stability of trivial equilibrium is $1+a_{2}+a_{1}+a_{0}>0$, i.e.

$1+a_{2}+a_{1}+a_{0}=1-p_{4}(1-h)-2hp_{2}p_{3}>0$.

Therefore,

$\left(1-p_{4}\right)/h+p_{4}>2p_{2}p_{3}$ ,

which suggests that the trivial equilibrium is locally unstable when

$\left(1-p_{4}\right)/h+p_{4}<2p_{2}p_{3}$ . (4)

ii) The stability of the non-trivial equilibrium of (B3)

The equilibrium (B3) exists when ineq. (B4) holds. The characteristic equation is

$$DetS_{1}=\left|\begin{matrix} 1-hx_{4}^{*}-hx_{4}^{*}(1-p_{4})-\lambda& (1-p_{1}) & (1-p_{2}) & (1-p_{3}) & (1-p_{4})(1-hx_{0}^{*})-hx_{0}^{*} \\ 0 & -\lambda& 0 & 0 & 0 \\ 2hx_{4}^{*} & p_{1} & -\lambda& 0 & 2hx_{0}^{*} \\ 0 & 0 & p_{2} & -\lambda& 0 \\ -hp_{4}x_{4}^{*} & 0 & 0 & p_{3} & p_{4}(1-hx_{0}^{*})-\lambda\end{matrix}\right|$$

$=(-\lambda)(1-\lambda)\left|\begin{matrix} 1 & 1 & 1 & 1 \\ 2hx_{4}^{*} & -\lambda& 0 & 2A \\ 0 & p_{2} & -\lambda& 0 \\ -hx_{4}^{*}p_{4} & 0 & p_{3} & p_{4}(1-A)-\lambda\end{matrix}\right|=(-\lambda)(1-\lambda)f(\lambda)=0$ , (B7)

where $f(\lambda)=\lambda^{3}+a_{2}\lambda^{2}+a_{1}\lambda+a_{0}=0$ and $a_{2}=-p_{4}(1-A)-hx_{4}^{*}(p_{4}-2)$, and $a_{0}=-2p_{2}\left\{hx_{4}^{*}(p_{4}-p_{3})+Ap_{3}\right\}$. The values of $a_{0}$ and $a_{1}$ are definitely negative because $x_{4}^{*},A>0$ and ineq. (B1). The value *a*_2_ can be rewritten as;

$$\begin{matrix} a_{2}=-p_{4}(1-A)-hx_{4}^{*}(p_{4}-2) \\ =-p_{4}(1-h)-p_{4}(h-A)-\frac{h-A}{1+2A+2p_{2}A}(p_{4}-2) \end{matrix}$$

$=-p_{4}(1-h)-\frac{2(h-A)}{1+2A+2p_{2}A}\left\{A(1+p_{2})p_{4}-(1-p_{4})\right\}$ .

As$A=\frac{1-p_{4}}{2p_{2}p_{3}-p_{4}}$, $A(1+p_{2})p_{4}-(1-p_{4})$ is proved to be positive because $p_{2}\leq p_{3}\leq p_{4}$. Therefore, *a*_2_ is negative as long as *A* and *h-A* are positive.

Corollary 1 in Appendix S1 tells us the necessary and sufficient condition of local stability is $1+a_{2}+a_{1}+a_{0}>0$.

Therefore, the equilibrium (B3) is locally stable if and only if

$\left(1-p_{4}\right)/h+p_{4}<2p_{2}p_{3}$. (B8)

Ineq. (B8) is the same as ineq. (B4). It means that the non-trivial equilibrium appears once ineq. (B8) is satisfied and, at the same time, it becomes locally stable and trivial equilibrium becomes unstable.

In evaluating the local stability, we ignored that one of the eigenvalues is equal to zero when the local stability is examined, because it comes from the fact that there occurs no colony with size 1. If the frequency of size-1 colony in the initial condition of eq. (1) is set as zero, the frequency is kept unchanged and the other variables move to the stable equilibrium. That is exactly what we expect to examine in the dynamics. We also ignored that one of the eigenvalues is equal to unity, because the sum of the frequencies is kept one in the dynamics of frequencies. It does not affect the local stability.
