# Supplementary material for: Ecological Conditions Favoring Budding in Colonial Organisms under Environmental Disturbance
Source: PLoS One. 2014 Mar 12;9(3):e91210. doi: 10.1371/journal.pone.0091210 (PMC3951312; doi:10.1371/journal.pone.0091210)
Supplement: Appendix S3 — Local stability analysis of positive equilibrium on the 1∶3 division strategy. (DOC) [file pone.0091210.s007.doc]

Supporting Information for " Ecological conditions favoring budding in colonial organisms under environmental disturbance," Mayuko Nakamaru, Takenori Takada, Akiko Ohtsuki, Sayaki, U. Suzuki, Kanan Miura, Kazuki Tsuji

Appendix S3 : Local stability analysis of positive equilibrium on the 1:3 division strategy

From eq. (2) in the main text, the dynamics of the 1:3 division strategy follows the difference equation as:

, (2)

or

.

There are two equilibria that satisfy

, (C1)

in the dynamics of Eq. (2). They are

,

and

, (C2)

where . The values of *B* and *hB-*1 should be positive for the existence of the non-trivial equilibrium and

. (C3)

The Jacobian matrix at the non-trivial equilibrium is

,

and the characteristic equation is

.

i) The stability of the trivial equilibrium,

The characteristic equation at the trivial equilibrium is

. (C4)

Therefore, we should examine the eigenvalues of

,

where , , and . Since and , Corollary 2 in Appendix S1 can be applied. The necessary and sufficient condition of local stability of the trivial equilibrium is $1+a_{3}+a_{2}+a_{1}+a_{0}>0$, i.e.

.

Therefore,

,

which suggests that the trivial equilibrium is locally unstable when

. (5)

When the trivial equilibrium is locally stable, non-trivial equilibrium does not exist because of eq. (C3).

ii) The stability of the non-trivial equilibrium of (C2)

The characteristic equation is

, (C5)

where , ,

*a*_2_ = −*hy*_4_^*^(2*p*_4_ − *p*_1_ − *p*_3_) − *hy*_0_^*^*p*_3_, and . The values of *a*_0_, *a*_1_ and *a*_2_ are definitely negative because and ineq. (B1). The value of *a*_3_ can be rewritten as;

.

The equation, , is proved to be positive because and . Therefore, *a*_3_ is negative as long as *B* and *hB–*1 are positive.

Since $a_{i}<0$, Corollary 2 in Appendix S1 can be applied to the necessary and sufficient condition of the local stability of the non-trivial equilibrium, which is , i.e.

.

Therefore,

. (C6)

Ineq. (C6) is the same as ineq. (C3). It means that the non-trivial equilibrium appears once ineq. (C6) is satisfied and, at the same time, it becomes locally stable and the trivial equilibrium becomes unstable.
