# Supplementary material for: Ecological Conditions Favoring Budding in Colonial Organisms under Environmental Disturbance
Source: PLoS One. 2014 Mar 12;9(3):e91210. doi: 10.1371/journal.pone.0091210 (PMC3951312; doi:10.1371/journal.pone.0091210)
Supplement: Appendix S4 — Local stability analysis in competitive system between two strategies. (DOC) [file pone.0091210.s008.doc]

Supporting Information for " Ecological conditions favoring budding in colonial organisms under environmental disturbance," Mayuko Nakamaru, Takenori Takada, Akiko Ohtsuki, Sayaki, U. Suzuki, Kanan Miura, Kazuki Tsuji

Appendix S4: Local stability analysis in competitive system between two strategies

in eq. (3) is expressed as

Here, *x_i_*(*t*) and *y_i_*(*t*) denote the densities of colonies of size *i* adopting the 2:2 and the 1:3 division strategies, respectively, and *z*_0_(*t*) denotes the density of empty sites (*z*_0_(*t*) +Σ*_i_* *x_i_*(*t*) + Σ*_i_* *y_i_*(*t*) = 1). Eq. (3) essentially comprises both Eq. (1) and (2). The exception is element (1, 1) in *F*(*w*(*t*)). This element, 1 - *hx*_4_(*t*) - *hy*_4_(*t*), embodies the assumption that no direct conflict arises between colonies adopting different dispersal strategies, but that colonies compete for empty sites.

When the growth probability is one (*g_i_* = 1), the dynamics of competitive system between two strategies for empty site is written by a 9 by 9 matrix, F, as:

, (D1)

where and

.

There are four equilibria that satisfy

, (D2)

in the dynamics of eq. (D1). They are

, ,

and

, (D3)

where and are the vectors whose elements are from the second to fifth elements of and in eqs. (B3) and (C2), respectively, and $x_{0}*$ and $y_{0}*$ are their first elements, respectively. (and $\mathbf{w}_{L}*$) exists when (B4) (and (C3)) is satisfied, respectively. and appear only when .

As shown in eq. (B5), the Jacobian matrix at equilibrium is

,

where .

i) The stability of the trivial equilibrium,

The characteristic equation at trivial equilibrium is

= 0. (D4)

The (2, 2) sub-matrix is the same as the matrix in eq. (B6) and the (3, 3) is the same as in Eq. (C4). Therefore, the stability condition of trivial equilibrium is and .

ii) The stability of

The characteristic equation is

DetC_S_ =

== 0,

(D5)

where $x_{i}*$ is the i-th element of in eq. (B3). The first determinant in eq. (D5) is identical with that in eq. (B7). The second is similar to that in eq. (C4) and *h* in eq. (C4) is replaced by in the second determinant. Therefore, the stability condition is inequalities (B8) and (5) where *h* is replaced by $hx_{0}^{*}$, i.e.

and . (4) and (6)

iii) The stability of

The characteristic equation is

DetC_L_ =

=

== 0,

(D6)

where $y_{i}*$ is the i-th element of in Eq. (C2). The first determinant of eq. (D6) is identical with that in eq. (C5). The second is similar to that in eq. (B6) and *h* in eq. (B6) is replaced by $hy_{0}^{*}$ in the second determinant. Therefore, the stability condition is inequalities (C6) and (4) where *h* is replaced by $hy_{0}^{*}$, i.e.

and . (5) and (7)

iv) The stability of

The value exists only when . The existence condition is a measure zero on the plane, and thus we stop discussing the stability condition of . We just point out that the equilibrium is not unique, and that the sum of and is constant: . It implies that the equilibrium of is a line on 9-dimensional space.
